# *O*-mannosylation of the *Mycobacterium tuberculosis* Adhesin Apa Is Crucial for T Cell Antigenicity during Infection but Is Expendable for Protection

**DOI:** 10.1371/journal.ppat.1003705

**Published:** 2013-10-10

**Authors:** Subhadra Nandakumar, Sunil Kannanganat, Karen M. Dobos, Megan Lucas, John S. Spencer, Sunan Fang, Melissa A. McDonald, Jan Pohl, Kristin Birkness, Venkateswarlu Chamcha, Melissa V. Ramirez, Bonnie B. Plikaytis, James E. Posey, Rama Rao Amara, Suraj B. Sable

**Affiliations:** 1 Division of Tuberculosis Elimination, National Center for HIV/AIDS, Viral Hepatitis, STD, and TB Prevention, Centers for Disease Control and Prevention, Atlanta, Georgia, United States of America; 2 Department of Microbiology and Immunology, Yerkes National Primate Research Center and Emory Vaccine Center, Emory University, Atlanta, Georgia, United States of America; 3 Department of Microbiology, Immunology and Pathology, College of Veterinary Medicine and Biomedical Sciences, Colorado State University, Fort Collins, Colorado, United States of America; 4 Biotechnology Core Facility Branch, Centers for Disease Control and Prevention, Atlanta, Georgia, United States of America; New Jersey Medical School, United States of America

## Abstract

Glycosylation is the most abundant post-translational polypeptide chain modification in nature. Although carbohydrate modification of protein antigens from many microbial pathogens constitutes important components of B cell epitopes, the role in T cell immunity is not completely understood. Here, using ELISPOT and polychromatic flow cytometry, we show that *O*-mannosylation of the adhesin, Apa, of *Mycobacterium tuberculosis (Mtb)* is crucial for its T cell antigenicity in humans and mice after infection. However, subunit vaccination with both mannosylated and non-mannosylated Apa induced a comparable magnitude and quality of T cell response and imparted similar levels of protection against *Mtb* challenge in mice. Both forms equally improved waning BCG vaccine-induced protection in elderly mice after subunit boosting. Thus, *O*-mannosylation of Apa is required for antigenicity but appears to be dispensable for its immunogenicity and protective efficacy in mice. These results have implications for the development of subunit vaccines using post-translationally modified proteins such as glycoproteins against infectious diseases like tuberculosis.

## Author Summary

*Mycobacterium tuberculosis* (*Mtb*) is the most devastating bacterial pathogen of all time that kills approximately 1.4 million people each year. *Mtb* modifies several of its proteins with sugar residues that influence many biological events. However, the significance of such sugar decorations and resulting carbohydrate and glycopeptide epitopes in shaping the T cell response during infection or after vaccination is insufficiently understood. Here, we show that the carbohydrate modifications of the *Mtb* Apa protein strikingly influence the magnitude of specific T cell responses in humans and mice after infection, but have only minor effect on the polyfunctionality and quality of T cell responses. The glycosylation of Apa was, however, expendable for T cell immunogenicity and protective efficacy when used either as a subunit vaccine or as a BCG-booster vaccine in dimethyl-dioctadecyl ammonium bromide (DDA)-monophosphoryl lipid A (MPL) adjuvant against virulent *Mtb* infection in mice. Our results suggest that the carbohydrate modification of microbial protein antigens may not always be critical for protection as our unmodified recombinant protein was sufficient for subunit vaccination. Together, our data underline the need to understand the role of heightened Apa glycoprotein-specific T cell responses in infection processes.

## Introduction

*Mycobacterium tuberculosis* (*Mtb*), the etiologic agent of tuberculosis (TB), produces an array of protein antigens (Ags), many of which are post-translationally modified [1]–[4], which constitute important determinants of innate and adaptive immune response. As with other pathogens, the post-translational protein modifications influence host interactions. In particular, carbohydrate modification of proteins serves as an efficient ligand for innate C-type lectin receptors (CLRs) present on the antigen presenting cells (APCs). Members of this receptor family play an important role in immune response induction, immune evasion, immune regulation and tolerance [5]. In addition to their role in innate immunity, carbohydrate modifications of protein Ags contribute to B cell epitopes, and it has been recently shown that glycopeptides may constitute a T cell epitope and can induce a strong T cell response [6]. Consequently, many glycoproteins or glyco-conjugates are considered Ags of interest in vaccine development. However, little is known about the role of protein glycosylation in T cell immunity in TB, and a better definition of immune responses to glycoproteins may aid in deciphering their role in protection or pathogenesis of *Mtb*.

The 45-47-kDa secretory and cell surface alanine-proline-rich antigen (Apa; Rv1860) is one of the few glycoproteins of *Mtb* for which the complete glycosylation pattern has been described [7], [8]. The modifications of native Apa (nApa) consist of complex *O*-mannosylation of specific threonine residues present at the N- and C-terminal domains [8], [9]. Apa is produced by all members of the *Mtb* complex including the vaccine strain, *M. bovis* bacillus Calmette Guerin (BCG). The nApa shares significant amino acid homology with a family of fibronectin attachment proteins found in other mycobacteria such as *M. avium, M. marinum* and *M. leprae* and is shown to possess fibronectin-binding activity [10]. In *Mtb*, mannosylated nApa is a potential adhesin and has a role in host cell attachment, entry, and immune evasion [11], [12].

The nApa is an immunodominant Ag and has been found to be strongly recognized by serum antibodies (Abs) of active TB patients [13], and the Ab reactivity was mainly directed against mannose residues. The Ag-specific T cell recognition of nApa also requires mannosylation. These mannose residues are absent in the recombinant form of Apa (rApa) expressed in *E. coli*, and the rApa is poorly recognized by T cells of BCG inoculated guinea pigs [14], [15]. We and others have identified Apa as a possible vaccine candidate against TB [16]–[18], but the role of glycosylation in inducing protective T cell immunity had not been studied.

Here, we investigated the effect of Apa mannosylation on the T cell antigenicity after mycobacterial infections and the immunogenicity and protective efficacy following protein vaccination. Antigenicity is the ability of the molecule to react with a preformed Ab or T cell receptor (TCR) while immunogenicity is the ability to elicit a cell-mediated or humoral immune response [19], which may or may not impart protection against a pathogen. Our results demonstrate that the mannosylation of Apa is critical for its T cell antigenicity in humans and mice after mycobacterial infections and provide evidence that a synthetic Apa glycopeptide constitutes a T cell Ag. We show that glycosylation of Apa is expendable for T cell immunogenicity and protective efficacy when used either as a subunit vaccine or as a BCG-booster vaccine against *Mtb* infection in BALB/c mice. Our data suggest that comparable immunogenicity and protective efficacy of mannosylated and nonmannosylated Apa forms may not be due to the inability of nApa to induce glycopeptide-specific T cells, as generation of nApa-specific hybridomas following subunit vaccination of mice identified a T cell clone specifically reactive to the N-terminal glycopeptide of nApa. Importantly, our data suggest that Apa may be considered a possible component of future vaccines against TB to boost waning BCG immunity, regardless of glycosylation.

## Results

### Apa Glycosylation and Responses of Human PBMCs

**Figure 1:**
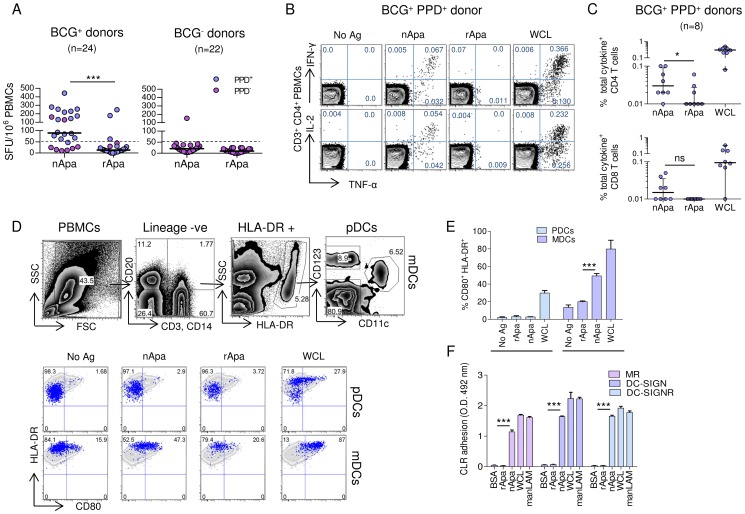
Human PBMC responses to nApa and rApa. (A–E) PBMCs from healthy donors were stimulated *in vitro* with no Ag, nApa, rApa or WCL, and the T cell or DC responses were investigated. (A) IFN-γ SFU/10^6^ PBMCs of BCG vaccinated (BCG^+^) and unvaccinated (BCG^−^) donors with (PPD^+^) or without (PPD^−^) reactivity to purified protein derivative, following stimulation with nApa or rApa. The median IFN-γ SFU/10^6^ PBMCs in no Ag stimulated wells (media only) were 7.5 (range 0–46.5) and 4.0 (range 0–37.5) for BCG^+^ and BCG^−^ donors, respectively. (B) The percentages (%) of TNF-α and IFN-γ (top) or IL-2 (bottom) producing cells among CD4^+^ T cells from 1 representative BCG^+^ PPD^+^ donor are shown, and (C) the frequency (%) of total cytokine producing cells among CD4^+^ (top) and CD8^+^ (bottom) T cells from 8 BCG^+^ PPD^+^ donors are plotted. (D) The % of CD123^+^ pDCs and CD11c^+^ mDCs expressing activation markers CD80 and HLA-DR in BCG^+^ PPD^+^ donor are shown (lower panel). The gating strategy for the two DC subsets is also depicted (upper panel). Similar results were obtained in BCG^+^ PPD^−^ donor. (E) Summary of pDCs and mDCs activation from 3 BCG^+^ donors regardless of PPD status. (F) Binding of nApa or rApa to recombinant human MR, DC-SIGN and DC-SIGNR *in vitro*. Data are representative of 2 independent experiments. Horizontal bars (A, C) and error bars (E, F) indicate the medians and means ± S.D., respectively. *Significant using 2-tailed Wilcoxon matched-pairs signed rank test (A and C) and by ANOVA (E, F).

We first determined the role of Apa mannosylation in T cell recognition and recall of cytokine responses in healthy, BCG vaccinated (BCG^+^) and BCG unvaccinated (BCG^−^) adults. The peripheral blood mononuclear cells (PBMCs) from 17/24 (70.8%) BCG^+^ individuals produced more than 50 IFN-γ spot forming units (SFU)/10^6^ cells (a positive response) after *in vitro* stimulation with purified nApa, as compared to those from only 3/24 (12.5%) individuals after stimulation with rApa (Figure 1A). Among BCG^+^ individuals, an IFN-γ response was predominantly observed in individuals with positive skin test reactivity to *Mtb* purified protein derivatives (PPD^+^), with 15/16 (93.7%) individuals showing nApa-specific positive response. Only 2/8 (25%) BCG^+^ individuals without PPD reactivity (PPD^−^) and 1/22 (4.5%) controls, i.e. BCG^−^PPD^−^ individuals, responded positively to nApa. This dominant nApa-specific T cell response was dose dependent, as demonstrated by the increased frequency of IFN-γ SFU when PBMCs of 5 healthy PPD^+^ (including 3 confirmed *Mtb*-exposed) individuals were stimulated with varying amounts of nApa, in contrast to different doses of rApa (Figure S1A). Overall, these results demonstrate that nApa is recognized preferentially over rApa in these individuals.

Next, we characterized the Ag-specific IFN-γ, TNF-α and IL-2 cytokine responses of 8 randomly selected BCG^+^PPD^+^ subjects by intracellular cytokine staining (ICS) and polychromatic flow cytometry and determined the percentages of CD4^+^ and CD8^+^ T cells expressing one (1+), any combination of two (2+), or all three (3+) cytokines. Significantly higher frequencies of IFN-γ, TNF-α or IL-2 producing CD4^+^ T cells were observed in nApa stimulated PBMCs than those stimulated with rApa (Figure 1B and 1C) and the response was characterized by higher percentages (but comparable proportions) of nApa-specific polyfunctional CD4^+^ T cells in the responding donors (Figure S1B). In contrast, the frequency of total cytokine producing CD8^+^ T cells was not statistically different after stimulation with the two forms of Apa (Figure 1C), although 4 donors had more nApa-specific cytokine producing CD8^+^ T cells. Collectively, these results suggest that mannosylation of Apa is important for the Ag-specific T cell recall responses in these individuals.

The heightened T cell responses seen with mannosylated Ags are presumably due to efficient uptake through mannose binding CLRs on the APCs such as dendritic cells (DCs) and higher efficiency of Ag presentation [20]–[22]. Therefore, we sought to determine whether Apa mannosylation influences its uptake by DCs. We found increased binding/uptake of FITC-labeled nApa as compared to labeled rApa by the blood monocyte derived dendritic cells (MoDCs) and by the CD11c^+^HLA-DR^+^ myeloid dendritic cells (mDCs) in the PBMCs of BCG^+^ and BCG^−^ individuals (Figure S1C and D). A higher percentage of myeloid (mDCs) but not plasmacytoid dendritic cells (pDCs) from human PBMCs expressed CD80 following *in vitro* stimulation with nApa than after rApa (Figure 1D and 1E), suggesting increased activation of mDCs by nApa. It is known that mannose binding CLRs such as DC-specific intracellular adhesion molecule-3 grabbing nonintegrin (DC-SIGN) and mannose receptor (MR) are highly expressed on mDCs but are absent on pDCs, and play an important role in the uptake and presentation of mannosylated Ags [23]. Corroborating these observations, we found that nApa but not rApa binds to recombinant human MR, DC-SIGN and DC-SIGNR using an *in vitro* CLR adhesion assay (Figure 1F).

To determine whether Ag presentation of nApa to specific T cells requires intracellular processing, APCs were fixed with glutaraldehyde before or after Ag pulsing. The MoDCs generated from the blood monocytes of three nApa-responding individuals (BCG^+^PPD^+^) were used as APCs, while T cells were purified from the PBMCs of respective individuals. The IFN-γ ELISPOT was used to investigate Ag presentation and activation of T cells after co-culture. Glutaraldehyde fixation of APCs before addition of nApa abrogated IFN-γ response (Figure S1E), whereas fixation of APCs after pulsing with nApa did not prevent IFN-γ production, although the response (SFU) was lower than pulsing of Ag alone without fixation. These results indicate a requirement for intracellular processing of nApa for presentation to T cells. In contrast, fixation of APCs before addition of the superantigen, staphylococcal enterotoxin B (SEB), did not prevent the IFN-γ response of T cells. Therefore, the lack of nApa-specific IFN-γ secretion by specific T cells, if fixation of APCs preceded Ag pulsing, is consistent with the presentation of cognate Ag (versus superantigen) [24], [25].

Next, to determine whether increased binding/uptake and activation of mDCs by nApa influences T cell reactivity and epitope recognition, we screened 32 sequential nonmodified synthetic overlapping peptides of Apa with PBMCs of BCG^+^PPD^+^ individuals who recognized both forms of Apa and those who recognized nApa over rApa. Several peptides induced positive IFN-γ responses in PBMCs from individuals who recognized both forms of Apa (Figure S1F), whereas no peptide induced >20 IFN-γ SFU/10^6^ PBMCs from individuals who recognized only nApa. These results suggest that mannosylation of Apa does not induce alternate Ag processing in APCs to produce unique peptide epitopes, but rather carbohydrate contributes to T cell epitopes and recognition of such mannosylated epitopes (for example glycopeptides) is probably responsible for elevated T cell responses to nApa in these individuals.

### nApa Is More Antigenic Than rApa in BCG or *Mtb* Infected Mice

**Figure 2:**
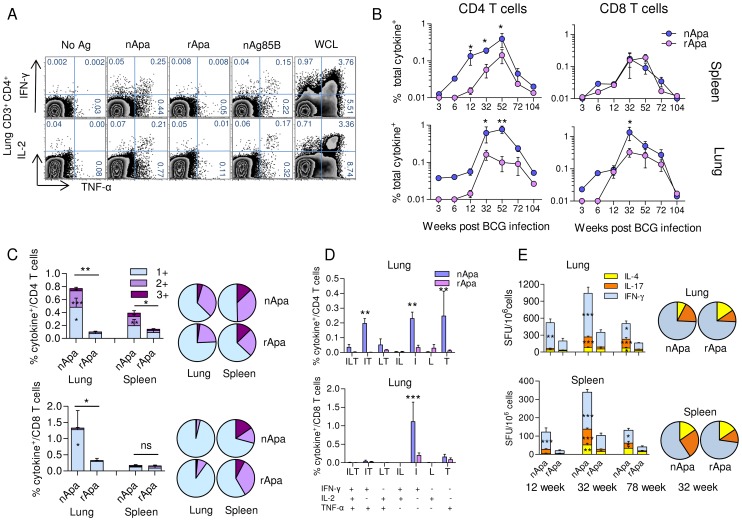
T cell responses to nApa and rApa in BCG infected mice. (A–E) Mice were infected s.c. with 1×10^6^ CFU BCG. At different time points after infection, as indicated, mice were euthanized (n = 4/time point) and their spleen or lung cells (pools) were stimulated *in vitro* with no Ag, nApa, rApa, nAg85B or WCL. (A) The percentages (%) of TNF-α and IFN-γ (top) or IL-2 (bottom) producing cells among lung CD4^+^ T cells from 1 representative experiment at the 52 wk time point are shown, and (B) the frequency (%) of nApa- or rApa-specific total cytokine producing cells among CD4^+^ and CD8^+^ T cells from the spleen and lung at 7 different time points encompassing entire life span are plotted. (C–D) nApa or rApa-specific cytokine co-expression profiles in the lung and spleen were determined, and (C) the proportions of single (1+), double (2+), or triple (3+) cytokine producing T cell subsets constituting total cytokine positive (+) CD4^+^ or CD8^+^ T cells at the peak of response are plotted as % of CD4^+^ T cells (top; at 52 wk) or CD8^+^ T cells (bottom; at 32 wk), respectively. The pie charts present the mean frequencies of 1+, 2+ and 3+ cytokine producers. (D) The percentages of 7 possible combinations of cytokine secreting subsets in the lung are also shown. Data at 12, 32, 52 and 72 wks (in B–D) are means ± s.e.m. of 3–4 independent mice experiments, while data (means) at 3, 6 and 104 wks are from one experiment evaluated in duplicate. Total as well as individual cytokine subset responses are compared. (E) nApa- or rApa-specific IFN-γ, IL-17 or IL-4 SFU/10^6^ lung or spleen cells at 12, 32 and 72 wks. Data are means ± s.e.m. of 2 independent mice experiments evaluated in triplicate. The pie charts present the mean IFN-γ, IL-17 and IL-4 SFU/10^6^ cells at 32 wk. *Significant using 1-way ANOVA followed by Bonferroni's multiple comparison test (B–E).

To determine whether Apa mannosylation is required for T cell antigenicity in animal models, BALB/c mice were infected with *Mtb* intranasally (i.n.) or with *M. bovis* BCG subcutaneously (s.c.). At different time points after infection, lung, spleen and draining inguinal lymph node (ILN) cells were isolated and stimulated with nApa or rApa. The frequency of IFN-γ, TNF-α and IL-2 producing CD4^+^ T cells after nApa stimulation of lung and spleen cells from BCG-administered mice was comparable (IFN-γ, IL-2) to 2-fold (TNF-α) more than that induced by nAg85B stimulation, whereas stimulation by rApa induced only a marginal increase in the frequency of cytokine secreting CD4^+^ T cells as compared to controls (Figure 2A and S2A). Similar results were also obtained using ILN cells (data not shown).

The time kinetics of CD4^+^ T cell response confirmed the presence of significantly higher frequency of nApa-specific total cytokine secreting cells in the lung (p = 0.0002) and spleen (p = 0.0021) as compared to those specific for rApa (Figure 2B) and revealed that the Apa responses peaked at 32–52 weeks (wks) after BCG administration, with a similar expansion and contraction trend for the nApa- and rApa-specific T cells. During the expansion phase, 3.9- and 8.6-fold more nApa-specific total cytokine secreting CD4^+^ T cells were observed at 12 wks in the lung and spleen, while at the peak (52 wks) the increase was 7.7- and 2.7-fold more in the lung and spleen, respectively. At individual cytokine levels, however, the difference between nApa- and rApa-specific cytokine producing CD4^+^ T cell frequency was more pronounced for IFN-γ and TNF-α (Figure S2B). These results suggest that mannosylation of Apa strongly influences the magnitude of Apa-specific CD4^+^ T cell responses in BCG administered mice and indicates that the CD4^+^ T cells of these mice predominantly recognize major histocompatibility complex-II (MHC-II) restricted epitopes present in nApa only. On the contrary, the frequency of nApa- and rApa-specific total cytokine secreting CD8^+^ T cells was not significantly different at the time points evaluated, except at the 32 wk time point in the lung (Figure 2B). This difference in the lung was mainly due to the presence of a higher frequency of nApa-specific IFN-γ producing CD8^+^ T cells (Figure S2C). These results indicate that both forms of Apa possess MHC-I restricted epitopes recognized by the CD8^+^ T cells of BCG administered mice.

Next, we compared the cytokine expression profiles of nApa- and rApa-specific CD4^+^ or CD8^+^ T cells. The lung CD8^+^ T cell response was dominated almost exclusively by 1+ cytokine (IFN-γ) producing cells (Figure 2C, histograms). In contrast, about 38% and 50% of the nApa-specific cytokine secreting CD4^+^ T cells produced more than 1 cytokine at the peak (52 wk) time point in the lung and spleen, respectively, and a significantly higher frequency of 2+ and 1+ cytokine producing CD4^+^ T cells was observed after stimulation with nApa than rApa in the lung (Figure 2C, histograms). When the Ag-specific cytokine producing lung CD4^+^ T cells were categorized into 7 distinct subpopulations based on cytokine expression profiles, IFN-γ and TNF-α co-producing cells dominated nApa-specific 2+ cytokine producing cells (Figure 2D), while IFN-γ or TNF alone producing cells were predominantly present among nApa-specific 1+ cytokine producers. Despite differences in the magnitudes, the proportions of nApa- and rApa-specific cytokine producing 3+, 2+ and 1+ cytokine producing CD4^+^ or CD8^+^ T cells was not significantly different in both organs at the peak time point (Figure 2C, pie charts).

The proportions of nApa- and rApa-specific IFN-γ, IL-4 and IL-17 SFU in cultured ELISPOT assay were also not significantly different when compared at 3 time points; however, statistically higher frequency of IFN-γ or IL-17 SFU was present in the lung and spleen cell cultures after nApa stimulation (Figure 2E). These results collectively suggest that Apa mannosylation may have only minor effect on the polyfunctionality and ratios of specific subpopulations of Apa-specific T cells in BCG inoculated mice. Similar dynamics were observed in *Mtb* Erdman infected mice (Figure S2D–F).

### Artificial Glycosylation of rApa C-terminus and T Cell Antigenicity

**Figure 3:**
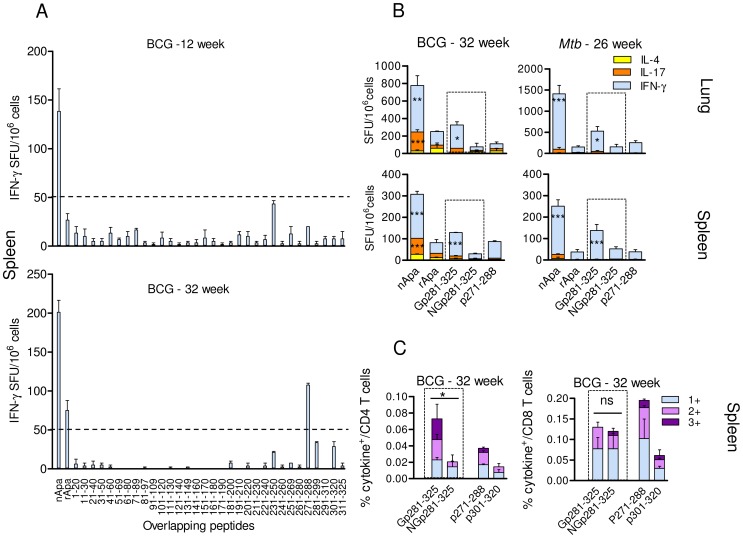
T cell reactivity to *in vitro*-mannosylated rApa C-terminal. (A) Pooled splenocytes from BCG infected mice (n = 4) were stimulated *in vitro* with nApa, rApa or 32 individual non-glycosylated synthetic Apa peptides in ELISPOT assay. IFN-γ SFU/10^6^ cells at 12 and 32 wk time points are shown. (B–C) Synthetic C-terminal glycopeptide (Gp281-325)- or control non-glycopeptide (NGp281-325)-specific T cell response (indicated inside dotted box) in mice. Stimulation with proteins (nApa or rApa) or non-glycopeptides (p271-288 or p301-320) was also included as controls. (B) Ag- or peptide-specific IFN-γ, IL-17 or IL-4 SFU/10^6^ pooled lung or spleen cells of BCG or *Mtb* infected mice at 32 and 26 wks, respectively. (C) The proportions of peptide-specific 1+, 2+ and 3+ cytokine producing T cell subsets constituting total cytokine positive (+) CD4^+^ T cells in the spleen of BCG infected mice at 32 wks, expressed as % of CD4^+^ T cells. Data in A–C are means ± s.e.m. *Significant using 1-way ANOVA followed by Bonferroni's test.

Thirty-two non-modified, synthetic overlapping peptides of Apa were screened for their capacity to induce IFN-γ response in mice at 12 and 32 wks post BCG infection. Only peptide p271-288 induced a positive response which only occurred 32 wks after infection (Figure 3A). These analyses support the indication that heightened T cell responses to nApa are mainly due to glycosylation. We synthesized rApa C-terminal peptide (residues 281–325; 45-mer) with di-mannosyl-threonine residue at position 316 (Fig. S3A), akin to that found in nApa, to confirm the role of Apa glycosylation in T cell antigenicity.

A higher frequency of IFN-γ SFU was observed in the spleen and lung cell cultures of BCG and *Mtb* infected mice after stimulation with the synthetic glycopeptide as compared to stimulation with the non-glycosylated control peptide (Figure 3B), confirming the role of Apa glycosylation in T cell antigenicity. A higher frequency of cytokine producing CD4^+^ T cells, but not CD8^+^ T cells, was found in the splenocytes of BCG mice after *in vitro* stimulation with glycopeptide compared to stimulation with control peptide (Figure 3C), suggesting that the carbohydrate modification of nApa C-terminus may constitute a CD4^+^ T cell epitope. The T cell epitope prediction analyses also indicated probable binding of a 15-mer encompassing Thr316 to MHC-II molecules (Figure S3B).

To determine whether T cells recognize carbohydrate (di-mannose) in the absence of the proper peptide context, we synthesized rApa C-terminal 45-mer peptide with di-mannosyl-threonine residue at its N-terminus (at an unnatural position) linked by the additional glycine residues (Figure S3C). We termed this Apa 45-mer with additional four N-terminal residues as a 49-mer glycopeptide (i.e., Apa non-glycopeptide residues 281–325 with Gly-Thr(di-man)-Gly-Gly extension). Significant cytokine response was observed in ELISPOT when the lung, spleen or ILN cells from the BCG mice were stimulated with the synthetic 45-mer glycopeptide with a di-mannosyl residue at its natural position (Thr316). On the contrary, no positive cytokine response was found after *in vitro* stimulation with the 49-mer glycopeptide, 49-mer nonglycopeptide (control) or the free di-mannose alone (Figure S3D). These results suggest that the carbohydrate (di-mannose) attached to the proper peptide backbone is likely required for the recognition of nApa C-terminal glycopeptide by specific T cells from BCG mice. Further characterization of MHC bound glycopeptide interactions with the TCR and the orientation of carbohydrate residues can be accomplished through crystallography studies.

### Both nApa and rApa Are Immunogenic Following Subunit Vaccination of Mice

**Figure 4:**
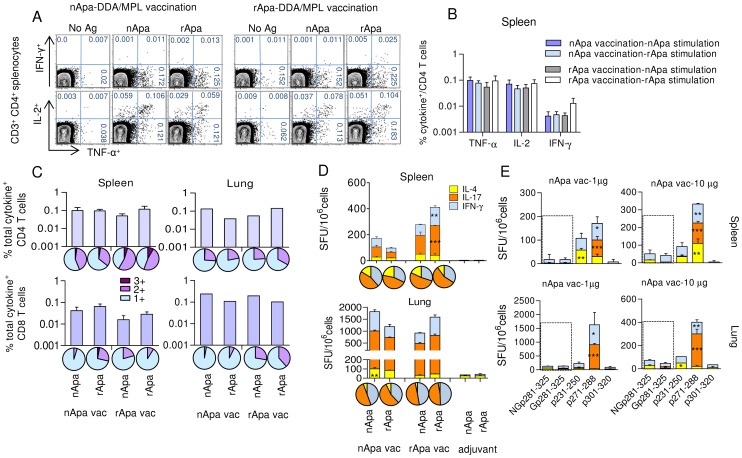
T cell responses in nApa or rApa vaccinated mice. (A–D) Mice were vaccinated with nApa or rApa in DDA-MPL adjuvant or with adjuvant alone (controls), and their spleen or lung cells were stimulated *in vitro* with no Ag, nApa or rApa 1 wk after last vaccination. (A) The percentages (%) of TNF-α and IFN-γ (top) or IL-2 (bottom) producing cells among splenic CD4 T cells of 1 representative mouse from nApa or rApa vaccinated group are shown, and (B) the frequency (%) of nApa- or rApa-specific individual cytokine producing cells among splenic CD4^+^ T cells from 4 mice/vaccinated group is plotted ( means ± s.e.m.). (C) The frequency (%) of nApa- or rApa-specific total cytokine producing cells among CD4^+^ (top) and CD8^+^ (bottom) T cells from the spleen (means ± s.e.m., n = 4 mice/group) and lung (pools) of nApa or rApa vaccinated (vac) mice. The corresponding pie charts present the mean frequencies of 1+, 2+ and 3+ cytokine producers. (D) The frequency of nApa- or rApa-specific IFN-γ, IL-17 or IL-4 SFU/10^6^ spleen or lung cells of vaccinated (vac) and control mice. The corresponding pie charts present the mean cytokine frequencies. Data are means ± s.e.m. of 3–4 wells using pooled cells. The responses of 2 Apa vaccinated groups were compared. *Significant by 1-way ANOVA followed by Bonferroni's test comparing respective immunogen-specific responses. (E) Synthetic Gp281-325- or NGp281-325-specific cytokine response of nApa vaccinated mice 4 wks post-vaccination. Stimulation with p231-250, p271-288 or p301-320 was evaluated for comparison.

To determine whether mannosylation of Apa also influences its ability to induce a protective immune response, mice were vaccinated s.c. with 3 doses of nApa or rApa (1 µg/dose) in the presence of dimethyl-dioctadecyl ammonium bromide (DDA) and monophosphoryl lipid A (MPL) adjuvants at 4 wk intervals, and the T cell responses were investigated 1 wk after the last dose. Stimulation of splenocytes or ILN cells of vaccinated mice with either nApa or rApa, resulted in a comparable frequency of individual cytokine producing CD4^+^ T cells, regardless of which form of the Ag was used for vaccination. This Apa-specific T cell response was characterized by more TNF-α and IL-2 than IFN-γ producing cells in an ICS assay (Figure 4A, 4B and S4A). The frequency of Ag-specific total cytokine producing CD4^+^ and CD8^+^ T cells induced in the spleen and lung by either Apa vaccine was also comparable (Figure 4C).

When the cytokine expression profiles of CD4^+^ T cells from the spleen and lung were evaluated in the vaccinated groups, IL-2 and TNF-α co-producing cells dominated 2+ cytokine producing cells, while TNF-α or IL-2 single-producers were predominantly present among 1+ cytokine producing cells (Figure 4A). In contrast, the cytokine expression profiles of CD8^+^ T cells in the spleen and lung were dominated by TNF-α or IFN-γ secreting 1+ cytokine producers, respectively. Higher proportions of total cytokine producing splenic CD4^+^ T cells of rApa vaccinated mice consisted of polyfunctional T cells than those of nApa vaccinated mice (Figure 4C). Otherwise, we found no significant difference in the quality of nApa- and rApa-specific response, when the proportions of 3+, 2+ and 1+ cytokine producing CD4^+^ and CD8^+^ T cells were determined in the two vaccinated groups. Significantly more immunogen-specific IL-17 and IFN-γ SFU were observed in the spleens of rApa vaccinated as compared to nApa vaccinated mice in cultured ELISPOT assay (Figure 4D, histograms). However, the proportions of specific IFN-γ, IL-17 and IL-4 SFU constituting the total ELISPOT response were not significantly different in nApa or rApa vaccinated mice (Figure 4D, pie charts).

Synthetic peptide screening in nApa and rApa vaccinated mice revealed that T cell cytokine responses were predominantly directed toward non-glycosylated p271-288 and p231-250 peptides (Figure S4B), regardless of the immunogen used, indicating that Apa mannosylation does not induce alternate Ag processing *in vivo* when administered in DDA-MPL. Next, we evaluated the lung and splenic T cell responses of C-terminal 45-mer glycopeptide (Gp-281-325) in nApa vaccinated mice. Comparable cytokine SFU after *in vitro* stimulation with 45-mer glycopeptide and its non-glycosylated control were found, regardless of dose of nApa (1 or 10 µg) used for vaccination (Figure 4E), and the p271-288-specific response was significantly more than that induced by Gp-281-325, collectively suggesting that the differences observed between nApa and rApa induced T-cell responses may be partially overcome by co-administration of appropriate adjuvants.

### Generation of nApa-Specific Hybridomas Identify N-terminal Glycopeptide-Reactive T Cell Clone

**Figure 5:**
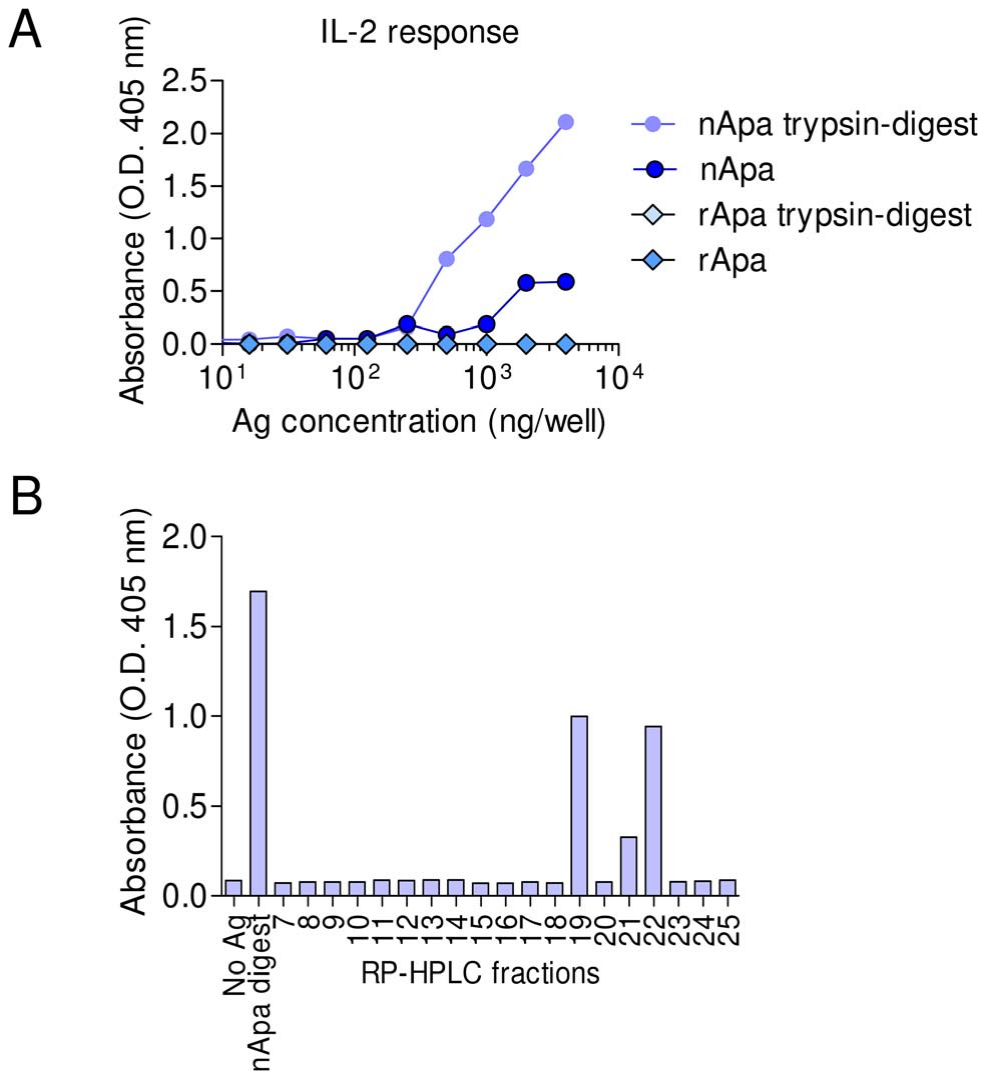
Characterization of nApa-specific T cell hybridoma clone (4C3). (A–B) The T cell hybridoma 4C3 was generated using draining LN cells of nApa-FIA vaccinated mice. The Ag and epitope specificity of 4C3 T cell clone was determined using IL-2 assay. (A) The 4C3 T cells (5×10^4^ cells/well) were cultured with APCs pulsed with indicated amounts of nApa, rApa or whole trypsin digests using T cell to APC ratio of 1∶1 for 24 h and culture supernatants were evaluated using IL-2 ELISA. (B) Identification of peptide/epitope specific for 4C3 T cell clone. The nApa was digested with trypsin and the peptide fragments were resolved by RP-HPLC, total 25 fractions were collected. Fractions 7–25 containing nApa peptides were used to screen the biological activity of 4C3 clone in IL-2 assay. The whole digest of nApa and fractions 19, 21, and 22 containing single N-terminal glycopeptide (residues p40-145) produced a positive IL-2 response when used for Ag pulsing of APCs. The fraction 19 is characterized by LC-MS (Figure S5B). The data (A and B) are means (O.D. 405 nm) of culture supernatant evaluations from two separate cultures in IL-2 ELISA. The experiment was repeated with similar results.

Since nApa is also glycosylated with complex mannose modifications at its N-terminus (Figure S3B), the presence of N-terminal glycopeptide(s)-specific T cells in our nApa vaccinated mice samples remained a possibility. Our preliminary attempt to synthesize and evaluate N-terminal glycopeptide (39-mer) was unsuccessful, and whether nApa glycopeptide-specific T cells are generated following subunit vaccination was not clear. Therefore, to identify nApa-specific T cell clones we employed a specialized protocol of T-cell hybridoma generation, using a single dose nApa vaccination of BALB/c mice in Freund's incomplete adjuvant (FIA). Individual T cell clones responding to nApa were purified by serial dilutions and tested for reactivity to nApa or rApa using an IL-2 capture ELISA after co-culture with APCs (i.e., syngeneic bone marrow derived dendritic cells) pulsed with Ag. Of the total 17 Apa-reactive hybridoma clones developed, 7 clones recognized both nApa and rApa while 10 clones recognized only nApa and not rApa (data not shown). Three of the 7 clones that recognized both nApa and rApa responded to a specific synthetic, nonmodified 15-mer peptide (two reactive to peptides spanning p271-288, and one to peptide p70-84, data not shown), while none of the 10 nApa reactive clones recognized any of the synthetic, overlapping, nonmodified peptides. Of the 10 clones only reacting to nApa, one clone, 4C3, reacted with a glycopeptide fraction of trypsin digested nApa fractionated by a reversed phase-HPLC column chromatography. Clone 4C3 produced significant amount of IL-2 when cultured with APCs pulsed with the whole digest of nApa but not with the digest of rApa (Figure 5A), indicating that the Ag presentation of nApa to 4C3 T cell clone was not inhibited by trypsin digestion. The RP-HPLC fractions consisting of the N-terminal 106 amino acid glycopeptide (residues p40-145) of nApa only demonstrated reactivity in IL-2 assay (Figure 5B, Figure S5A and S5B). Glycosylation of the N-terminus of nApa from *Mtb* likely explains specific recognition of the 4C3 clone to the nApa peptide. The lack of biological activity of T cell clone 4C3 when presented with rApa or synthetic nonglycosylated peptides collectively suggest that N-terminal glycopeptide-specific T cells are generated after nApa subunit vaccination. Further confirmation of these results will require identification of the precise epitope and synthesis of the N-terminal glycopeptide.

### nApa or rApa Vaccination Imparts Comparable Protection against *Mtb* Challenge

**Figure 6:**
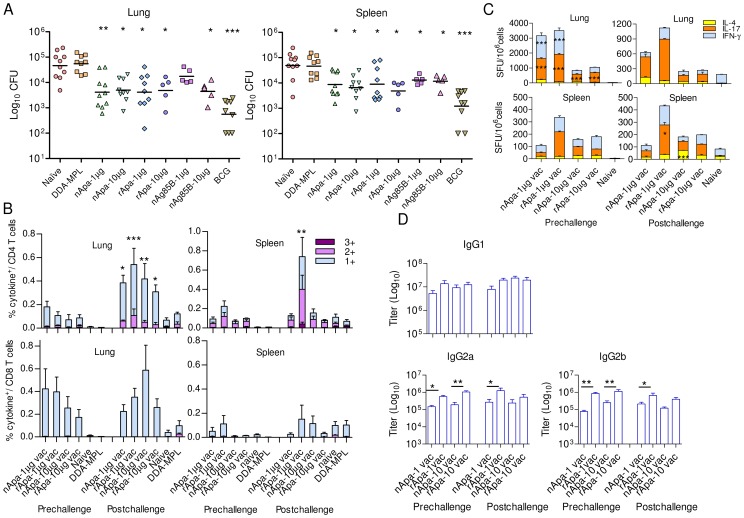
Protective potential of Apa against *Mtb* challenge and recall of T and B cell responses. (A) Protective efficacy of nApa or rApa vaccinated mice using 1 or 10 µg/dose in DDA-MPL against *Mtb* challenge. Twelve wks after first vaccine injection, mice were challenged with virulent *Mtb* Erdman. The differences in bacterial load between vaccinated and nonvaccinated control groups 6 wks after challenge are shown. Data are using 5–10 mice per group. (B–C) T cell responses of Apa vaccinated and control mice pre- and post-challenge. Respective immunogen (i.e. Apa form used for vaccination)-specific responses in Apa vaccinated (vac) groups while nApa-specific responses in control groups are shown. (B) The proportions of 1+, 2+, or 3+ cytokine producers (of IFN-γ, TNF-α and IL-2) constituting Apa-specific total cytokine positive (+) CD4^+^ or CD8^+^ T cells are plotted as % of CD4^+^ T cells (top) or CD8^+^ (bottom) T cells in the lungs and spleen of Apa vaccinated and control mice. Data are means ± s.e.m. of 4 mice/group. (C) The frequency of Apa-specific IFN-γ, IL-17 or IL-4 SFU/10^6^ pooled spleen or lung cells of vaccinated and control mice. Data are means ± s.e.m. of triplicate cultures. (D) Immunogen-specific IgG1, IgG2a and IgG2b antibody titers in the sera of Apa vaccinated mice. Data are means ± s.e.m. of 4 mice/group. Data in A, B and D are representative of 2 independent experiments. *Significant by Kruskal-Wallis followed by Dunn's test comparing vaccinated group with naïve and adjuvant controls in protection experiment (A), by 1-way ANOVA followed by Bonferroni's test comparing pre- and post-challenge responses of respective vaccinated groups (B and C) or comparing indicated groups (D).

We investigated the protective efficacy of two Apa forms using DDA-MPL, a known Th1 adjuvant. BALB/c mice were vaccinated s.c. with nApa or rApa 3-times at 4 wk intervals using two different concentrations (1 or 10 µg), in DDA-MPL adjuvant and challenged with *Mtb* 4 wks after the last dose to assess their protective potential. Mice vaccinated with nAg85B, a known protective Ag, were included for comparison. Mice immunized once with live BCG at the start of vaccination were used as positive controls, while negative controls received adjuvant alone (3-times) or were left untreated (naïve). Both forms of Apa imparted significant protection compared to naïve and adjuvant control groups at 2 different doses used (Figure 6A), and the bacterial burden among the Apa vaccinated groups was not significantly different. Vaccination with nAg85B also induced protection, but only nAg85B-10 µg was statistically different from the negative control groups at the level of lung and spleen. However, the degree of protection afforded by BCG vaccination was greater than any of the subunit vaccinated groups. Vaccination with either nApa or rApa induces comparable levels of protection in our model and suggests that glycosylation of Apa is not critical for protection.

### Apa Vaccination and Ability to Recall T and B Cell Responses after Challenge

To ascertain whether comparable protective efficacy offered by nApa and rApa vaccination in mice correlated with an equal ability to recall cellular and humoral immune responses after *Mtb* challenge, T cell responses and serum Ab titers were measured before *Mtb* challenge (at 4 wks after last vaccination) and 6 wks post challenge. All Apa vaccinated groups presented with an equal vaccine immunogen-specific CD4^+^ T cell recall response in the lungs after challenge, characterized by a significantly higher frequency of total cytokine producing CD4^+^ T cells in the post-challenge versus pre-challenge group (Figure 6B). Significant recall from spleen derived T cells was only observed in rApa-1 µg group. No significant differences were observed when the immunogen-specific lung or spleen CD8^+^ T cell responses were compared between vaccine immunogens; however, the CD8^+^ T cell responses of all vaccinated groups were higher than those of control groups in the lungs. Both nApa and rApa were recognized equally well when used for stimulation of lung and spleen cells of vaccinated and challenged groups (Figure S6A). Comparable patterns of cytokine co-expression profiles were found when T cell responses of respective Apa vaccinated groups were compared before and after challenge (Figure 6B); characterized by immunogen-specific TNF-α and IL-2 producing 2+ and 1+ CD4^+^ T cells in the spleen and IFN-γ producing 1+ CD4^+^ and CD8^+^ T cells in the lungs. On the contrary, splenic CD4^+^ T cells responses of the challenged naïve controls were characterized by nApa-specific IFN-γ and TNF-α producing 1+ and 2+ CD4^+^ T cells only.

The cultured ELISPOT assay revealed that the frequency of immunogen-specific total IFN-γ, IL-17 and IL-4 SFU in the lung was significantly lower in vaccinated-challenged groups compared to respective vaccinated group pre-challenge (Figure 6C). These results suggest possible decrease in *in vitro* proliferation of immunogen-specific lung T cells in ELISPOT cultures after recent *in vivo* recall or regulatory responses suppressing cytokine release following *Mtb* challenge. Proof for any of these explanations will require further in-depth investigation. All Apa vaccinated groups produced comparable amounts of immunogen-specific serum IgG1 Abs before and after challenge (Figure 6D), and these Abs recognized nApa and rApa equally well (Figure S6B). However, rApa vaccination induced significantly higher amounts of IgG2a and IgG2b Abs than nApa vaccination, except at 10 µg dose post-challenge.

**Figure 7:**
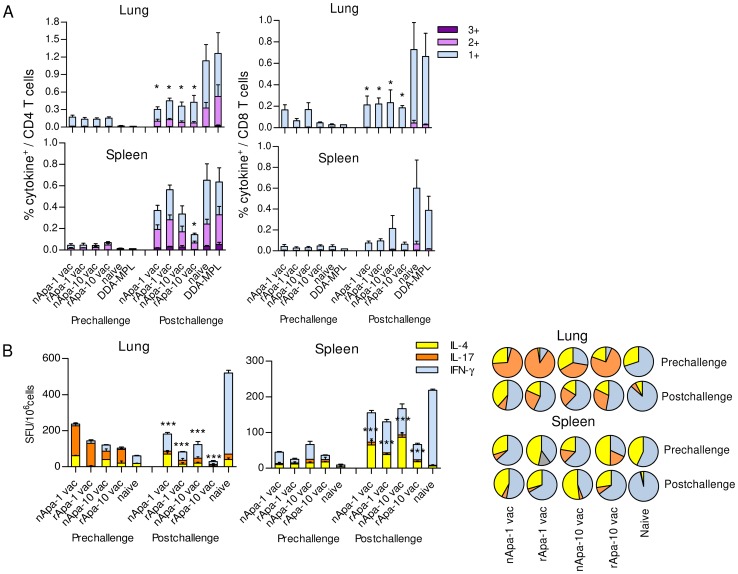
Pathogen-specific responses in Apa vaccinated and control mice after challenge. (A) The proportions of STCF-specific 1+, 2+ and 3+ cytokine producers (of IFN-γ, TNF-α and IL-2) constituting STCF-specific total cytokine positive (+) CD4^+^ or CD8^+^ T cells are plotted as % of CD4^+^ T cells or CD8^+^ T cells in the lung and spleen of Apa vaccinated and control mice. Data are means ± s.e.m. of 4 mice/group. (B) The frequency of STCF-specific IFN-γ, IL-17 or IL-4 SFU/10^6^ pooled spleen or lung cells of vaccinated and control mice. Data are means ± s.e.m. of 3 wells. The corresponding pie charts present mean cytokine frequencies. *Significant by 1-way ANOVA followed by Bonferroni's test comparing responses of each vaccinated group with adjuvant control group post-challenge.

Of importance, when pathogen-specific immune responses were investigated in the lung and spleen of vaccinated and control groups of mice *in vitro*, higher frequency of *Mtb* short term culture filtrate (STCF)-specific total cytokine producing CD4^+^ and CD8^+^ T cells was observed in naïve and adjuvant only mice as compared to vaccinated mice post-challenge (Figure 7A), with a significant difference at the level of lung. As expected, STCF-specific responses of unvaccinated-challenged mice were dominated by IFN-γ and TNF-α 1+ and 2+ CD4^+^ T cells and IFN-γ 1+ CD8^+^ T cells. Furthermore, when STCF-specific IFN-γ, IL-17 and IL-4 SFU were compared among challenged groups, responses of unvaccinated mice were found to be highly skewed toward IFN-γ (87–96% of total response), with significantly more SFU compared to all vaccinated groups (Figure 7B).

### Apa Glycosylation and Boosting BCG-Induced Immunity

**Figure 8:**
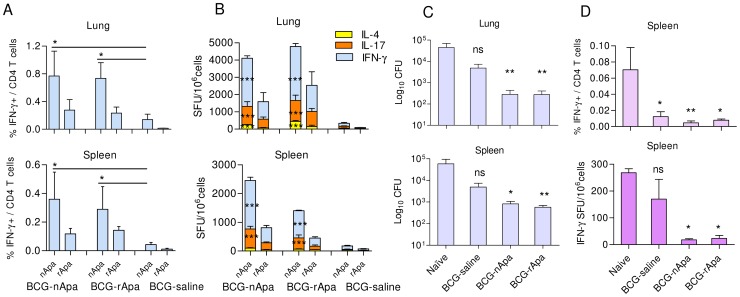
BCG booster potential of Apa. (A–D) Mice were vaccinated s.c. with 1×10^6^ CFU BCG and 16 months later boosted twice with either saline alone or nApa or rApa (1 µg/dose) in DDA-MPL by same route. (A) Percentages (%) of nApa- or rApa-specific IFN-γ producing cells among lung or spleen CD4^+^ T cells of 3 groups of mice were determined 5 wks after last booster dose. Data are means ± s.e.m. of 4 mice/group. (B) The frequency of nApa- or rApa-specific IFN-γ, IL-17 or IL-4 SFU/10^6^ pooled lung or spleen cells are plotted. *Significant by 1-way ANOVA followed by Bonferroni's test comparing nApa-specific responses of saline booster group with those of Apa-boosted groups. (C) Protective efficacy of nApa, rApa or saline boosted BCG mice against *Mtb* challenge was determined. The differences in bacterial load between BCG boosted and age-matched naïve mice (n = 5–6/group), 6 wks post-challenge are shown. (D) ESAT-6+CFP-10-specific IFN-γ response in the spleens of mice was determined as a surrogate for bacterial burden. Data are mean ± s.e.m. of 4 mice/group for CD4^+^ T cell response (top) and mean ± s.e.m. of triplicate cultures for total SFU response (bottom). *Significant by Kruskal-Wallis test followed by Dunn's test, ns- non-significant with respect to age matched naïve controls.

Considering the fact that most individuals in the world have been vaccinated with BCG and new TB vaccines have to be considered in the context of prior BCG vaccination, development of strategies aimed at boosting and improving the protective immunity induced by BCG is considered to be one of the rational approaches to develop an effective vaccination regimen against TB. Since BCG induced protection wanes significantly by 18 months in mice (unpublished data), we investigated the booster effect of 2 doses of nApa or rApa administered 3 wks apart in DDA-MPL at 16 months after BCG vaccination. Both forms of Apa significantly boosted T cell responses compared to boosting with saline, and a significantly higher frequency of T cells from the Apa-boosted groups recognized nApa over rApa after *in vitro* stimulation (Figure 8A and B). Nonetheless, comparable nApa-specific responses were found in both Apa-boosted groups, except for splenic IFN-γ SFU. Of importance, boosting with either form of Apa, but not with saline, significantly reduced *Mtb* CFU in the lung and spleen of BCG mice compared to age-matched controls (Figure 8C). BCG mice boosted with nApa or rApa showed about 2.0-log reduction in bacterial counts in the lungs as compared to age-matched naïve mice, while 1.7-log to 2.0-log reduction was found in the spleens of nApa- and rApa-boosted mice, respectively. This protection is characterized by a significantly lower frequency of ESAT-6+CFP-10-specific total as well as CD4^+^ T cell IFN-γ responses in the spleens of Apa-boosted groups (Figure 8D).

## Discussion

Glycosylation generates an enormous variety of modified protein-derived epitopes. However, the significance of such modified antigenic epitopes in intracellular bacterial pathogens in shaping the T cell repertoire and determining the outcome of an immune response is poorly understood. Here we demonstrate that mannosylation of *Mtb* Apa (also known as MPT-32 or ModD) is crucial for its T cell antigenicity during mycobacterial infections of humans and mice and provides direct evidence that bacterial mannopeptides are T cell Ags using a synthetic Apa glycopeptide. Previously, glycopeptides containing tumor associated carbohydrates [26], [27], glycopeptides of self-Ags in autoimmune diseases [28], and artificially glycosylated peptides of model Ags [6] have been shown to be recognized by T cells. In antigenic glycopeptides, the peptide backbone usually provides the binding motif that interacts with the MHC molecule, while the glycan provides an important part of the structure (epitope) that is recognized by the T cell receptor [29]. Conversely, pure carbohydrates are usually incapable of MHC binding and T cell stimulation, and due to their haptenic nature requires a carrier. The exception to this immunologic paradigm is zwitterionic polysaccharides which are presented in an MHC class-II restricted manner [30]. Because complex carbohydrates are not removed during processing by DCs, it remains possible that mannosylated peptide epitopes within nApa are recognized *in vitro* by T cells primed in infected mice and humans [31]. Our synthetic peptide screenings suggest that the recognition of carbohydrate containing epitopes (i.e., glycopeptides) rather than increased Ag uptake, mDCs activation or alternate Ag processing leading to enhanced recognition of other peptide epitopes is the major determining factor for heightened T cell responses to nApa during infection. Mapping of the precise mannosylated epitopes will further help to identify whether mannose binds to the MHC groove along with peptide backbone or interacts strictly with TCR and will provide direct insight into the role of mannosylation in Ag presentation of Apa to T cells.

Unexpectedly, vaccination of mice with nApa or rApa induced comparable CD4^+^ or CD8^+^ T cell frequency, regardless of the form of Apa used for *in vitro* stimulation. Nonetheless, these data together with post-challenge recall responses revealed that the paucity of anti-rApa T cell responses after primary infection, especially CD4^+^ T cell responses, is not due to an intrinsic inability of rApa to induce these responses. However, why nApa is recognized highly discriminately by T cells during infection but not after subunit vaccination remains to be answered. Speculatively, mannosylated epitope(s) of nApa might be effectively generated in phagosomes during intracellular growth of BCG or *Mtb in vivo*, but the relative dominance of mannose bearing epitopes versus peptide epitopes may change during processing of nApa in the presence of DDA-MPL in the endo-lysosomes of APCs *in vivo* following subunit vaccination. It is known that transport to different types of compartments in APC can lead to differential processing and generation of different epitopes within the same protein [32] and that different T cell epitopes in the same protein have been found to be recognized during infection and after subunit vaccination [32]. In our protocol, the majority of specific T cells generated after nApa-DDA-MPL vaccination might have been directed against the dominant peptide epitopes (such as p271-288 and p231-250) and the frequency of glycopeptide-specific T cells, if at all generated, might have been diminished in nApa stimulated *in vitro* cultures. The carbohydrate or glycopeptide epitope-specific T cells induced by glycoprotein vaccination have been reported to be scarce and highly sensitive to *in vitro* culture conditions [6], [33]. The requirement of specialized protocols for their expansion [33] including generation of T cell hybridomas has been suggested. Generation of nApa-specific T cell hybridomas following subunit vaccination of mice using FIA identified a T cell clone (4C3) specifically responding to the glycosylated N-terminal peptide of nApa. The dominant nature of peptide epitope p271-288 was also confirmed, because two hybridomas (3F7 and 2D10) that recognized both nApa and rApa strongly recognized synthetic peptides spanning p271-288 in IL-2 assay (data not shown). Therefore, the comparable immunogenicity of nApa and rApa following subunit vaccination may not be due to the inability of nApa to induce glycopeptide-specific T cells.

Of paramount importance, both nApa and rApa offered comparable protection against virulent *Mtb* challenge in mice, regardless of whether the 1 or 10 µg/dose was used for vaccination. Despite higher *in vitro* immunogen-specific cytokine SFU induced in the lungs by 1 µg than 10 µg/dose vaccination and the subtle differences in the quality of serum Ab response induced by the two forms of Apa vaccination prechallenge, comparable protection was found in all Apa vaccinated groups. Therefore, mannosylation appears to be dispensable for protective efficacy of Apa in DDA-MPL adjuvant in our model of progressive TB. It remains to be seen whether heightened T cell responses can be induced against nApa than its recombinant counterpart by using a different delivery vehicle or adjuvant system and whether the immunogenicity and protective efficacy of nApa differs from rApa in FIA, the adjuvant used for T cell hybridoma generation. However, immunization with a mannosylated model peptide in Freund's complete adjuvant (FCA) has been previously shown to induce poor Th1 effector functions despite enhanced Ag presentation [34]. Nonetheless, the ability of rApa to impart protection in this study is consistent with previous observation that i.n. subunit vaccination using rApa protein imparts notable protection in mice [18].

Of note, a comparable protection was offered by nAg85B and nApa vaccination despite the presence of 10 to 30-fold higher magnitude of immunogen-specific lung CD8^+^ and CD4^+^ T cells in nAg85B vaccinated mice (data not shown). In addition, WCL-specific T cell responses induced by BCG in the lung prechallenge were 2 to 5-fold lower than the immunogen-specific responses induced by nAg85B vaccination, but, the protection imparted by nAg85B was not better than BCG. These results suggest that no direct correlation exists between the magnitude of immunogen-specific T cell responses induced by subunit vaccination and the degree of protection afforded against *Mtb* challenge. Previously we have shown that the *Mtb* proteins selected on the basis of dominant T cell and IFN-γ response during human infection do not necessarily impart stronger protection in vaccination experiments [35]–[36]. Interestingly, the decreased T cell responsiveness with higher dose (10 µg) Apa vaccination did not adversely affect the protection using either form in challenge experiments. Understanding the mechanism of such immune regulation and Apa vaccination induced protection will require further investigation.

One of the important findings in this study is the particularly significant protective efficacy of Apa when used as a BCG-booster vaccine against *Mtb* challenge in the elderly mice with waning BCG induced protective immunity. Stronger and comparable protection observed in the nApa or rApa-boosted BCG mice, whose T cell responses are mainly primed against mannose modifications of nApa, further suggests that mannosylation of Apa is dispensable for BCG-boosting potential. It is conceivable that a similar effect might be seen in humans previously vaccinated with BCG in childhood and whose immunity has waned, although of course, this would require clinical verification.

The progress of glycoproteins as vaccine candidates often suffers a serious roadblock during the developmental pipeline. It is usually due to perceived loss of immunogenicity when produced as a recombinant protein, synthetic peptides or DNA vaccine due to complete absence or lack of appropriate glycosylation. Despite effectiveness of several glyco-conjugate vaccines against extracellular pathogens, it has been suggested that artificial glycosylation of peptides and targeting of CLRs may not always lead to generation of protective immunity [37], as CLRs are also targeted by pathogens to evade host immune responses [5], [38]. Furthermore, some ligand-MR interactions can result in promotion of Th2, anti-inflammatory or regulatory responses and may even lead to T cell anergy and tolerance [34], [39]. Glycosylation can mask protective epitopes in Ag, prevent effective Ag processing, inhibit proteolysis by masking cleavage sites and shift the pattern of peptides processed by DCs [40], [41]. Contrarily, tolerance induction or CLR engagement can be a disease defense strategy [42], [43] and it is argued that a mere ‘Th1 vaccine’ employing conserved immunodominant Ags can induce hyper-immune activation and may be advantageous to *Mtb* [44] and a complex balance between pro- and anti-inflammatory host factors is required to prevent and control *Mtb* infection [45]. Our results, therefore, underscore the importance of conducting comparative protection studies in animal models with a glycosylated Ag and its non-glycosylated counterpart. This may prevent elimination of poorly antigenic but otherwise protective non-glycosylated candidate during preclinical evaluation and may help save lengthy, costly and complex developmental efforts to artificially glycosylate it, carried out with the aim to improve its immunogenicity.

In summary, we provide the evidence that the natural glycosylation of a protein may differentially affect its antigenicity and immunogenicity- the two attributes known to influence the protective efficacy of TB subunit vaccines [19], [46]. Although Apa mannosylation influences T cell antigenicity during infection, it is expendable for induction of protective immunity following vaccination. Recently, a protein-*O*-mannosylating enzyme has been found to be required for virulence of *Mtb* [47] and our results highlight the need for further investigation of the role of increased mannosylated epitope-specific T cells in infection. In view of the finding that *Mtb* Apa improves waning BCG immunity and imparts significant protection in elderly mice, it makes a strong case for its inclusion as a possible component of future vaccines against TB.

## Materials and Methods

### Ethics Statement

The study was approved by the Institutional Review Board of Centers for Disease Control and Prevention (CDC), Atlanta, Georgia, USA, (approved protocol number 1652), and informed written consent was obtained from all human participants before withdrawal of venous blood. All animal experiments performed in BSL-II or BSL-III animal facilities were in strict accordance with the guidelines of the U.S. Public Health Service Policy on the Humane Care and Use of Animals and the Guide for the Humane Care and Use of Laboratory Animals. The Institutional Animal Care and Use Committees of Centers for Disease Control and Prevention, Atlanta, Georgia, and the Colorado State University, Fort Collins, Colorado, USA reviewed and approved these animal protocols (approval numbers SABMOUC1664, 1847, SHIMOUC 1490 and CSU 98-026A).

### Antigens

The nApa was purified from *Mtb* H37Rv culture filtrate by traditional and reversed-phase chromatography as described previously [8]. The recombinant clone for Apa (pMRLB.17), WCL, STCF, nAg85B, rESAT-6 and rCFP-10 of *Mtb* H37Rv were obtained through the NIH Biodefense and Emerging Infection Research Resources Repository. The rApa was expressed in *E. coli* BL21 (DE3) and purified from lysates by Nickel chromatography with endotoxin removal using analogous methods described elsewhere [48] followed by DEAE-Sepharose chromatography. The overlapping peptides of Apa and the C-terminal 45-mer and 49-mer (glyco) peptides were synthesized by the Fmoc/tbu solid-phase peptide synthesis strategy [49], [50]. See Supplementary Methods (Text S1) for details.

### Human Subjects

Blood was obtained from 50 healthy adult individuals (26 BCG^+^ and 24 BCG^−^). All donors were HIV negative and were without any clinical signs of TB. All BCG^+^ donors received BCG as a neonatal vaccine. We excluded responses of 1 BCG^−^ and 2 BCG^+^ donors who tested positive for hepatitis. All 16 BCG^+^ donors with positive tuberculin skin test reactivity (>10 mm induration after intradermal injection) had normal chest radiographs. However, 1 BCG^−^ and 4/16 BCG^+^ donors demonstrated positive reactivity to ESAT-6+CFP-10 in PBMC ELISPOT assay. The *Mtb* exposure of these donors was further confirmed by commercial IFN-γ ELISPOT (Oxford Immunotech) and ELISA (Cellestis) tests (IGRAs). Remaining 22 BCG^−^ donors had negative tuberculin skin test and no known history of contact with individuals with TB. The exposure of donors to environmental mycobacteria is not known but all live in Atlanta area with very low incidence of atypical mycobacterial infections. All donors demonstrated reactivity to phytohaemagglutinin (PHA) in ELISPOT assay and none were anergic.

### Vaccinations and Experimental Infections of Mice

Specific-pathogen-free, 6–8 wk old female BALB/c (H-2^d^) mice (Harlan Sprague Dawley) were used in the study. BCG vaccinated mice received 100 µl of 1×10^6^ CFU Copenhagen s.c. above the gluteus superficialis and biceps femoralis muscles of hind legs (50 µl/leg). For protein vaccination, groups of mice received 200 µl of nApa, rApa or nAg85B (1 or 10 µg) in DDA and MPL (from *Salmonella minnesota* Re 595) [51] s.c. on hind legs (100 µl/leg) either 3-(subunit) or 2-times (prime-boost vaccination). *Mtb* infections were performed i.n. with 5×10^4^ CFU Erdman strain. At 48 h after *Mtb* challenge, about 0.5% of the total CFU delivered could be cultured from the lungs. The bacterial burden was determined by plating organ homogenates onto Middlebrook 7H10 agar supplemented with OADC and TCH (2 µg/ml). CFU were enumerated after 4 wks of incubation at 37°C.

### ELISPOT Assay

IFN-γ, IL-4 or IL-17A ELISPOT assay was performed using commercially available human or mouse ELISPOT reagent set (BD-Biosciences) according to the manufacturer's protocol as described previously [18], except that no DCs were added as supplemental APCs and cells were stimulated with 10 µg/ml of purified or complex *Mtb* Ags, synthetic peptides or di-mannose (unless mentioned otherwise) for 40 h in RPMI-1640. See Supplementary Methods (Text S1) for details.

### Flow Cytometry

Expression of cell surface markers and intracellular cytokine production by human PBMCs or mouse organ cells after *in vitro* stimulation with *Mtb* Ags (10 µg/ml) at 37°C for total 12 h was assessed as described previously [52]. See Supplementary Methods (Text S1) for details.

### Binding/Uptake Assay

For fluorescent labeling of proteins, commercially available FITC labeling kit (Pierce Biotechnology) was used. The protein labeling was carried out in 50 mM sodium borate buffer (pH 8.5) using 2 mg/ml protein concentration. Unreacted excess florescent dye was removed using spin columns with purification resin as per the manufactures instructions. Protein concentration was assessed using the BCA assay (Sigma). The efficiency of labeling was determined by Nano Drop Fluorospectrometer and was comparable between nApa and rApa. Protein samples were stored in the dark at −20°C before use. Human blood monocyte derived DCs (MoDCs) were generated as described before [53] using recombinant human GM-CSF (80 ng/ml) and IL-4 (40 ng/ml) (PeproTech). Freshly isolated human PBMCs or MoDCs (day 6) were pulsed with FITC-labeled nApa, rApa or WCL (20 µg/ml) and incubated at 4°C or 37°C for the indicated amounts of time. Cells were washed with PBS containing 10% FBS. Samples were then stained with desired Abs to identify DC subsets and analyzed using flow cytometry.

### CLR Adhesion Assay

*Mtb* nApa or rApa was coated onto ELISA plates (Nunc, Maxisorp) at 5 µg/well in 0.1 M carbonate-bicarbonate buffer (pH 9.6); and coating took place for 18 h at room temperature. Wells coated with 5 µg/well of purified BSA fraction-V (Fisher Scientific) were used as negative controls while those coated with *Mtb* H37Rv WCL or mannose caped LAM served as positive controls. Blocking was carried out with 1% BSA for 30 min at 37°C and additional 1.5 h at room temperature in TSM buffer (20 mM Tris-HCL (pH 7.4) containing 150 mM NaCl, 2 mM CaCl_2_ and 1 mM MgCl_2_) [54]. Soluble recombinant human DC-SIGN (CD209)-Fc chimera, DC-SIGNR (CD299)-Fc chimera or MR (CD206) (R&D Systems) (2.5 µg/ml in TSM buffer) was added and the adhesion was performed for 2 h at room temperature. Unbound CLR was washed away and the binding of DC-SIGN-Fc or DC-SIGNR-Fc was determined by an anti-IgG1-Fc ELISA using a peroxidase conjugate of goat anti-human-Fc. Binding of MR was determined by anti-human-MR (CD206) mouse mAb (clone 685641; R&D Systems) followed by peroxidase conjugate of anti-mouse IgG. The plates were developed using *o*-phenylenediamine dihydrochloride (Sigma-Aldrich) and absorption was measured at 492 nm. Specificity of CLR binding was determined by blocking the interaction in the presence of either 2 mg/ml mannan or 5 mM EDTA (OD 492 nm <0.1).

### Ag Processing and Presentation Assay

The PBMCs were isolated from the venous blood of healthy BCG^+^PPD^+^ human donors who responded to nApa in IFN-γ ELISPOT assay. Recombinant human GM-CSF and IL-4 developed MoDCs (day 6) were used as APCs while T cells purified from the PBMCs of respective donors using ‘Dynabeads Untouched Human T Cells’ purification kit (Invitrogen) and magnetic separation (>95% pure) were used as effector cells. APCs (2×10^6^) were pulsed with either nApa (10 µg/ml) or SEB (1 µg/ml) or without any Ag (complete RPMI-1640 media only) and incubated at 37°C. After 4 h, APCs were washed with RPMI-1640 and were either left untreated or fixed with 0.05% glutaraldehyde for 5 min at 22°C. APCs were washed and the fixation was stopped by incubation with 0.2M glycine in RPMI-1640 at 22°C. After 5 min, cells were washed 4 times with RPMI-1640. Alternatively, APCs were fixed and Ag pulse was carried out for 4 h. APCs were washed 4 times after Ag pulse and were co-cultured with purified T cells (1∶2 ratio) in ELISPOT plates pre-coated with anti-human IFN-γ capture Abs. After 40 hr of incubation at 37°C in the presence of 5% CO_2_, ELISPOT plates were developed and SFU were counted.

### Generation of nApa-Specific T Cell Hybridomas and IL-2 Assay

T-cell hybridomas specific for *Mtb* nApa were generated as previously described [55]. Briefly, four BALB/c mice were each vaccinated with 40 µg of nApa in FIA by injecting 25 µl into each hind footpad and the remainder at the base of the tail. Five days after the vaccination, the draining lymph nodes (popliteal, inguinal and periaortic) were harvested to obtain lymph node (LN) cells. The primed LN cells were restimulated *in vitro* with syngeneic bone marrow derived dendritic cells (BMDCs) that had been pulsed with 10 µg/ml nApa per well. Approximately 1×10^6^ primed LN cells were added per well in two 24-well tissue culture plates in a total volume of 1 ml of complete RPMI 1640 medium (RPMI 1640 supplemented with 10% FCS, 5×10^−5^M 2-ME (Sigma), plus a nutrient cocktail as described [56]). After two days of culture, the cells were harvested and pooled from all 48 wells, washed, fused with the T cell fusion partner BWα^−^β^−^ [57], and plated out into ten 96-well plates. Clones that grew in individual wells were screened using either BMDCs, as above, or a BALB/c mouse-derived B cell lymphoma line, A20 [58], pulsed with 0.5 µg/well nApa. The selection of responding T cell hybridomas was performed by assaying 24 hr culture supernatants using paired rat monoclonal Abs specific for mouse IL-2 (Pharmingen/BD-Biosciences) in a capture ELISA. For Ag presentation studies, microculture wells were prepared containing 250 µl of culture medium, 5×10^4^ each of T cell hybridoma and APC, and a known amount of nApa or rApa (intact or trypsin digested), in flat-bottomed 96-well microtiter wells. Dose response curves with native and recombinant Ags were performed to determine which T cell hybridomas responded nApa alone, or to both the native and recombinant form of the protein. T cell hybridomas were further tested with a panel of synthetic, overlapping, nonglycosylated Apa peptides or trypsin digested and RP-HPLC separated nApa fractions to determine the peptide epitopes that were recognized. For details regarding trypsin digestion of Apa and characterization of RP-HPLC fractions please refer to Supplementary Methods (Text S1).

### Ab ELISA

ELISA assays of mouse sera were performed as described previously [46], using 2 µg/ml of nApa or rApa for coating and HRP-conjugated anti-mouse IgG1, IgG2a (BD-Pharmingen) and IgG2b (Santa Cruz Biotech) Abs for detection.

### Statistical Analyses

Differences between groups were assessed by the parametric Student's *t* test or 1-way ANOVA followed by Bonferroni's test and the nonparametric 2-tailed Mann-Whitney *U*-test (the Wilcoxon test for matched pairs) or Kruskal-Wallis followed by the Dunn's post-test (GraphPad Prism program). Unless indicated, all immune response data are presented after subtracting no Ag control values. A value of p<0.05 was considered to be significant and * <0.05; ** <0.01; *** <0.001.

### Accession/Identification Numbers

The accession/identification numbers of *Mtb* proteins used in the study are *Mtb* (H37Rv) alanine and proline-rich antigen (Apa), Rv1860, NCBI reference sequence: YP_177849.1; *Mtb* (H37Rv) Ag85B, Rv1886c, NCBI reference sequence: NP_216402.1; *Mtb* (H37Rv) 6 kDa early secretory antigenic target (ESAT-6), Rv3875, NCBI reference sequence: YP_178023.1; *Mtb* (H37Rv) 10 kDa culture filtrate protein (CFP-10), Rv3874, UniProtKB/Swiss-Prot reference sequence: P0A566.2.

## Supporting Information

Figure S1 **Human PBMC responses to nApa and rApa.** (A) IFN-γ response kinetics of purified nApa and rApa in the PBMCs of healthy PPD^+^ donors with (+) or without (−) BCG vaccination and ESAT-6 and CFP-10 reactivity, following *in vitro* stimulation with increasing amounts of Ag (0.1 to 10 µg/ml) in ELISPOT assay. Each symbol represents an individual donor, while the horizontal bar indicates a median response. (B) Cytokine expression profiles of nApa and rApa- specific CD4^+^ T cells. PBMCs from PPD^+^ (n = 8) or PPD^−^ (n = 3) BCG vaccinated donors were stimulated with nApa, rApa or WCL (10 µg/ml) and IFN-γ, IL-2 and TNF-α cytokine co-expression profiles were determined using the Boolean gating. The proportions of subsets of CD4^+^ T cells positive for one (1+), any combination of two (2+), or all three (3+) cytokines constituting total cytokine positive (+) Ag-specific CD4^+^ T cells were determined for each donor and expressed as percentages of CD4^+^ T cells and plotted as histograms. The pie charts present the mean frequencies of single (1+), double (2+) and triple (3+) cytokine producers of BCG^+^PPD^+^ donors specific for each Ag. (C–D) Uptake of nApa and rApa by DCs. The PBMCs from the healthy BCG^+^PPD^+^ or BCG^−^PPD^−^ individuals or MoDCs from the BCG^+^PPD^+^ individuals (n = 3) were pulsed with indicated FITC-labeled Ags for 2 h and Ag uptake was analyzed by flow cytometry. (C) A histogram gated on CD11c^+^HLA-DR^+^ cells is shown from one representative experiment using PBMCs from BCG^+^PPD^+^ individual and pulsed with FITC-nApa (dark blue histogram) and FITC-rApa (light blue histogram) for 2 h at 37°C. Background uptake of nApa-FITC after 2 h at 4°C is shown (white histogram). (D) Summary of FITC-labeled nApa and rApa uptake by CD11c^+^HLA-DR^+^ blood DCs and MoDCs *in vitro*. The MFI is represented by the geometric mean of the gated CD11c^+^HLA-DR^+^ peak. The data are means ± s.e.m. of three independent experiments. *Significant using 1-way ANOVA followed by Bonferroni's test. (E) Processing and presentation of nApa to T cells. APCs (MoDCs) from nApa responding BCG^+^PPD^+^ individuals (n = 3) were fixed with glutaraldehyde before or after pulsing with no Ag, nApa or SEB for 4 h at 37°C. A set of APCs were also pulsed without fixing for 4 h. After incubation APCs were washed and cultured with T cells purified from the PBMCs of the respective individuals for 40 h in IFN-γ ELISPOT plates. The data are mean ± s.e.m SFU/10^6^ purified T cell from 3 individuals evaluated in duplicate cultures. TNTC- too numeric to count. (F) Synthetic peptide screenings. The frequency of nApa, rApa or 32 individual non-modified synthetic Apa peptides-specific IFN-γ secreting cells in the PBMCs of BCG^+^PPD^+^ donors recognizing both nApa and rApa or those recognizing nApa much better than rApa was determined by ELISPOT assay and expressed as mean SFU/10^6^ PBMCs.
(TIF)

Figure S2 **nApa is more antigenic than rApa in BCG and** ***Mtb*** **infected mice.** (A–C) Mice were infected s.c. with 1×10^6^ CFU BCG, or (D–F) i.n. with 5×10^4^ CFU *Mtb*. At different time points after infection, as indicated, mice were euthanized and their spleen or lung cells were stimulated *in vitro* with no Ag, nApa, rApa, nAg85B or WCL. (A) The percentages (%) of TNF-α and IFN-γ (top) or IL-2 (bottom) producing cells among spleen CD4^+^ T cells from one representative experiment at the 52 wk time point are shown, and (B) the frequency (%) of nApa- or rApa-specific IFN-γ, IL-2 or TNF-α producing cells among CD4^+^ and (C) IFN-γ producing cells among CD8^+^ T cells from the spleen and lung at 7 different time points are plotted. Data at 12, 32, 52 and 72 wks (in B–C) are means ± s.e.m. of 3–4 independent mice experiments, while data (means) at 3, 6 and 104 wks are from one experiment using pooled cells (n = 4 mice) evaluated in duplicate. (D–F) T cell response in *Mtb* infected mice. (D) nApa- or rApa-specific IFN-γ, IL-2 and TNF-α cytokine co-expression profiles in the spleen and lung were determined at 12 and 26 wks after infection and the proportions of single (1+), double (2+), or triple (3+) cytokine producing T cell subsets constituting total cytokine positive (+) CD4^+^ or CD8^+^ T cells are plotted as % of CD4^+^ T cells (right) or CD8^+^ T cells (left), respectively. Total as well as individual subset responses are compared. (E) The percentages of 7 possible combinations of cytokine secreting CD4^+^ or CD8^+^ T cell subsets in the lung at 26 wks are also plotted. Data at 12 wks are means ± s.e.m. of pooled cell culture in triplicate while at 26 wks are of individual mice (n = 4). (F) nApa or rApa-specific IFN-γ, IL-17 or IL-4 SFU/10^6^ spleen or lung cells at 26 wks. Data are means ± s.e.m. of triplicate or quadruplet cultures. * Significant using 1-way analysis of variance (ANOVA) followed by Bonferroni's multiple comparisons test (B–F).
(TIF)

Figure S3 **Amino acid sequence of** ***Mtb*** **Apa and synthetic C-terminal glycopeptides and antigenicity.** (A) Structure of a synthetic 45-mer C-terminal Apa glycopeptide (residues 281–325). (B) Amino acid sequence of Apa. The signal peptide and the sequence of the protein are indicated in italic and bold-faced type, respectively. Parentheses indicate amino acid 137, phenylalanine for *Mtb*, and leucine for BCG. The sequence of synthetic 45-mer C-terminal glycopeptide and control non-glycopeptide (residues 281–325) evaluated in this study is indicated in the box. Threonine (Thr) residues at positions 50 and 57 are naturally glycosylated with mannobiose (α-D-Manp(1→2)α-D-Manp), the Thr residue at position 66 is modified with a single mannose (α-D-Manp), and Thr-316 is glycosylated with either a mannose, a mannobiose, or a mannotriose (α-D-Manp(1→2)α-D-Manp(1→2)α-D-Manp) (indicated in red color). The T cell epitope prediction analysis using IEDB program (http://www.immuneepitope.org/) indicated the probable binding of a 15-mer (residues 309–323) encompassing Thr316 to MHC class-II molecules encoded by H2I-A^d^ and H2E-A^d^ alleles, while peptide p271-288 is predicted to consist of 2 overlapping 9-mers with high affinity for MHC class-I molecules encoded by H2K^d^ allele (IEDB and http://www.syfpeithi.de/). The black solid line, dotted line and blue line indicates IEDB program predicted CD8^+^, CD4^+^ and B-cell epitopes (based on Kolaskar and Tongaonkar antigenicity) in Apa C-terminal domain (residues 271–325), respectively. (C) Structure of a synthetic 49-mer glycopeptide comprised of Apa peptide (residues 281–325, non-glycosylated at Thr-316) and N-terminal extension bearing dimannosyl-Thr residue linked to Apa 45-mer sequence via Gly-Gly dipeptide unit. (D) Synthetic 45-mer glycopeptide-, 49-mer glycopeptide- and free di-mannose-specific cytokine responses of BCG infected mice in ELISPOT assay. Stimulation with mature proteins (nApa or rApa) or non-glycopeptides (45-mer and 49-mer) was included as controls. Ag- or peptide-specific IFN-γ, IL-17 or IL-4 SFU/10^6^ pooled lung, spleen or ILN cells of BCG infected mice at 58 weeks are shown. Data are means ± s.d. *Significant using 1-way ANOVA followed by Bonferroni's test.
(TIF)

Figure S4 **Immunogenicity of nApa and rApa in mice.** (A) Mice were vaccinated with nApa or rApa (1 µg/dose) in DDA-MPL adjuvant and 1 wk after last vaccination their ILN cells (pools of 4 mice/group) were stimulated *in vitro* with nApa or rApa. The frequency (%) of nApa- or rApa-specific TNF-α, IL-2 or IFN-γ cytokine producing cells among CD4^+^ T cells is plotted. (B) Synthetic peptide screenings. The frequency of nApa-, rApa- or 32 individual non-modified synthetic Apa peptides-specific IFN-γ, IL-17 or IL-4 cytokine secreting cells in the spleen of 10 µg/dose nApa or rApa vaccinated mice was determined 1 wk after last dose using ELISPOT assay and expressed as mean SFU/10^6^ cells ± S.D. Similar peptide recognition pattern was observed after 1 µg/dose vaccination.
(TIF)

Figure S5 **Fractionation of nApa trypsin-digest and LC-MS analysis of fractions.** (A) The RP-HPLC fractionation of nApa trypsin-digest. A chromatogram with relative abundance of each fraction is shown. (B) LC-MS analysis of nApa trypsin-digest. A portion of each fraction was analyzed by LC-MS to identify the peptides represented by each fraction. Fraction 19 (shown), 21, and 22 (not shown) demonstrated the presence of the N-terminal glycopeptide for nApa, mannosylated with 5 (predominant) 4, or 3 residues. No other significant products were found in the most active fractions (19 and 22). The amino acid sequence of the N-terminal nApa glycopeptide is shown in the box.
(TIF)

Figure S6 **Both nApa and rApa are recognized equally well in Apa vaccinated and challenged mice.** (A–B) Mice were vaccinated with nApa or rApa (either 1 or 10 µg/dose) in DDA-MPL adjuvant. Four wks after last vaccination dose, mice were challenged with *Mtb* as described in Figure 5. T and B cell responses to both nApa and rApa were investigated at the time of challenge and 6 wks post-challenge. (A) The frequency (%) of TNF-α, IL-2 or IFN-γ cytokine producing cells in the lung and spleen cells of Apa (1 µg/dose) vaccinated and challenged mice after *in vitro* stimulation with nApa or rApa are shown. Data are average response of 4 mice/group (± s.e.m.). (B) Anti-nApa and anti-rApa IgG1, IgG2a and IgG2b antibody titers in the sera of Apa vaccinated mice before and after challenge. Data are average response of 4 mice/group (± s.e.m.).
(TIF)

Text S1 **Supplementary information.** Supplemental information includes details of peptide synthesis, ELISPOT assay, flow cytometry, trypsin proteolysis of Apa and RP-HPLC fractionation of digests, and Table S1.
(DOCX)
