# Supplementary figures and images for: O-mannosylation of the Mycobacterium tuberculosis Adhesin Apa Is Crucial for T Cell Antigenicity during Infection but Is Expendable for Protection
Source: PLoS Pathog. 2013 Oct 10;9(10):e1003705. doi: 10.1371/journal.ppat.1003705 (PMC3795050; doi:10.1371/journal.ppat.1003705)

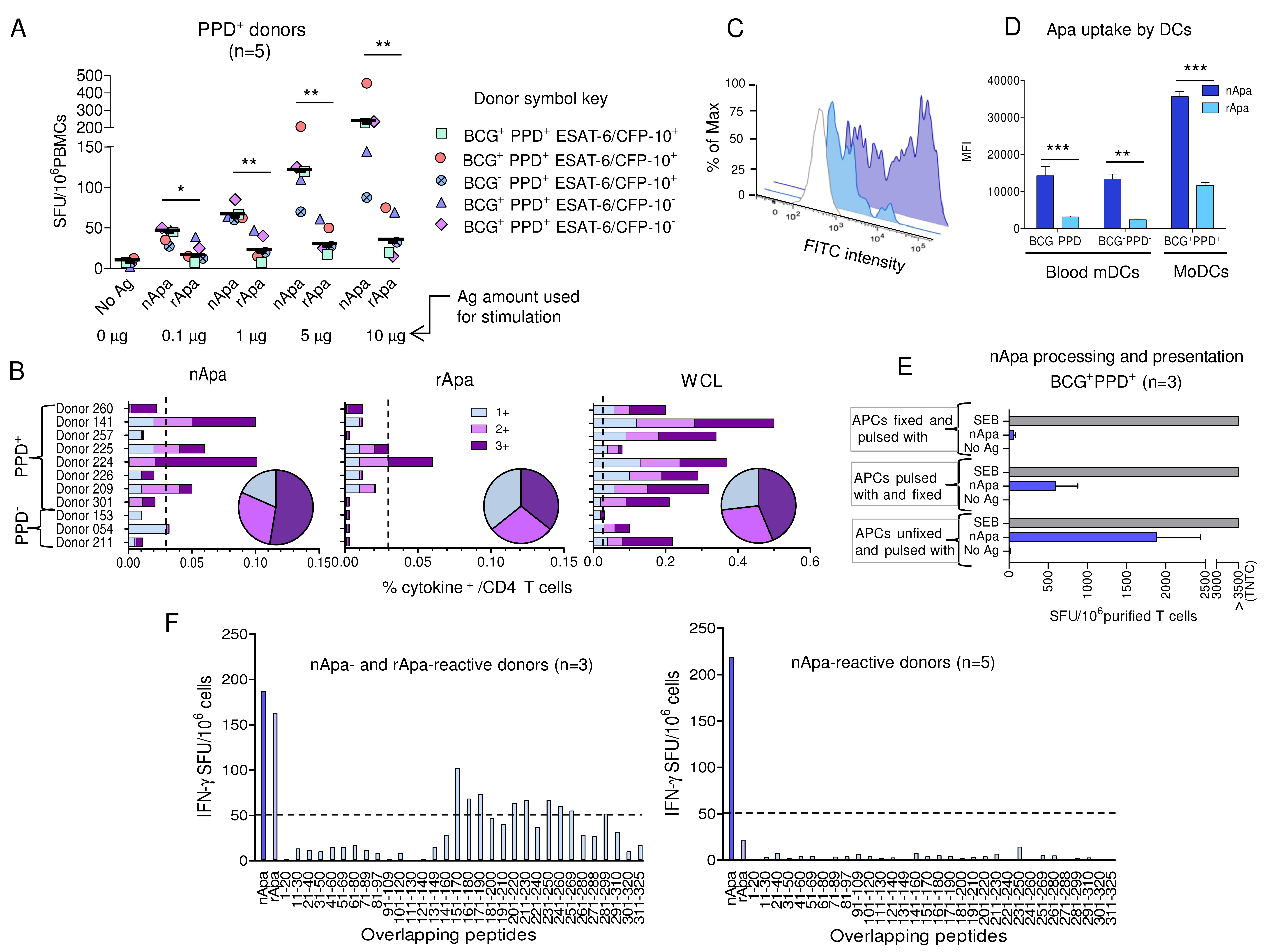

Supplement: Figure S1 — Human PBMC responses to nApa and rApa. (A) IFN-γ response kinetics of purified nApa and rApa in the PBMCs of healthy PPD+ donors with (+) or without (−) BCG vaccination and ESAT-6 and CFP-10 reactivity, following in vitro stimulation with increasing amounts of Ag (0.1 to 10 µg/ml) in ELISPOT assay. Each symbol represents an individual donor, while the horizontal bar indicates a median response. (B) Cytokine expression profiles of nApa and rApa- specific CD4+ T cells. PBMCs from PPD+ (n = 8) or PPD− (n = 3) BCG vaccinated donors were stimulated with nApa, rApa or WCL (10 µg/ml) and IFN-γ, IL-2 and TNF-α cytokine co-expression profiles were determined using the Boolean gating. The proportions of subsets of CD4+ T cells positive for one (1+), any combination of two (2+), or all three (3+) cytokines constituting total cytokine positive (+) Ag-specific CD4+ T cells were determined for each donor and expressed as percentages of CD4+ T cells and plotted as histograms. The pie charts present the mean frequencies of single (1+), double (2+) and triple (3+) cytokine producers of BCG+PPD+ donors specific for each Ag. (C–D) Uptake of nApa and rApa by DCs. The PBMCs from the healthy BCG+PPD+ or BCG−PPD− individuals or MoDCs from the BCG+PPD+ individuals (n = 3) were pulsed with indicated FITC-labeled Ags for 2 h and Ag uptake was analyzed by flow cytometry. (C) A histogram gated on CD11c+HLA-DR+ cells is shown from one representative experiment using PBMCs from BCG+PPD+ individual and pulsed with FITC-nApa (dark blue histogram) and FITC-rApa (light blue histogram) for 2 h at 37°C. Background uptake of nApa-FITC after 2 h at 4°C is shown (white histogram). (D) Summary of FITC-labeled nApa and rApa uptake by CD11c+HLA-DR+ blood DCs and MoDCs in vitro. The MFI is represented by the geometric mean of the gated CD11c+HLA-DR+ peak. The data are means ± s.e.m. of three independent experiments. *Significant using 1-way ANOVA followed by Bonferroni's test. (E) Processing and presentation [file ppat.1003705.s001.tif]

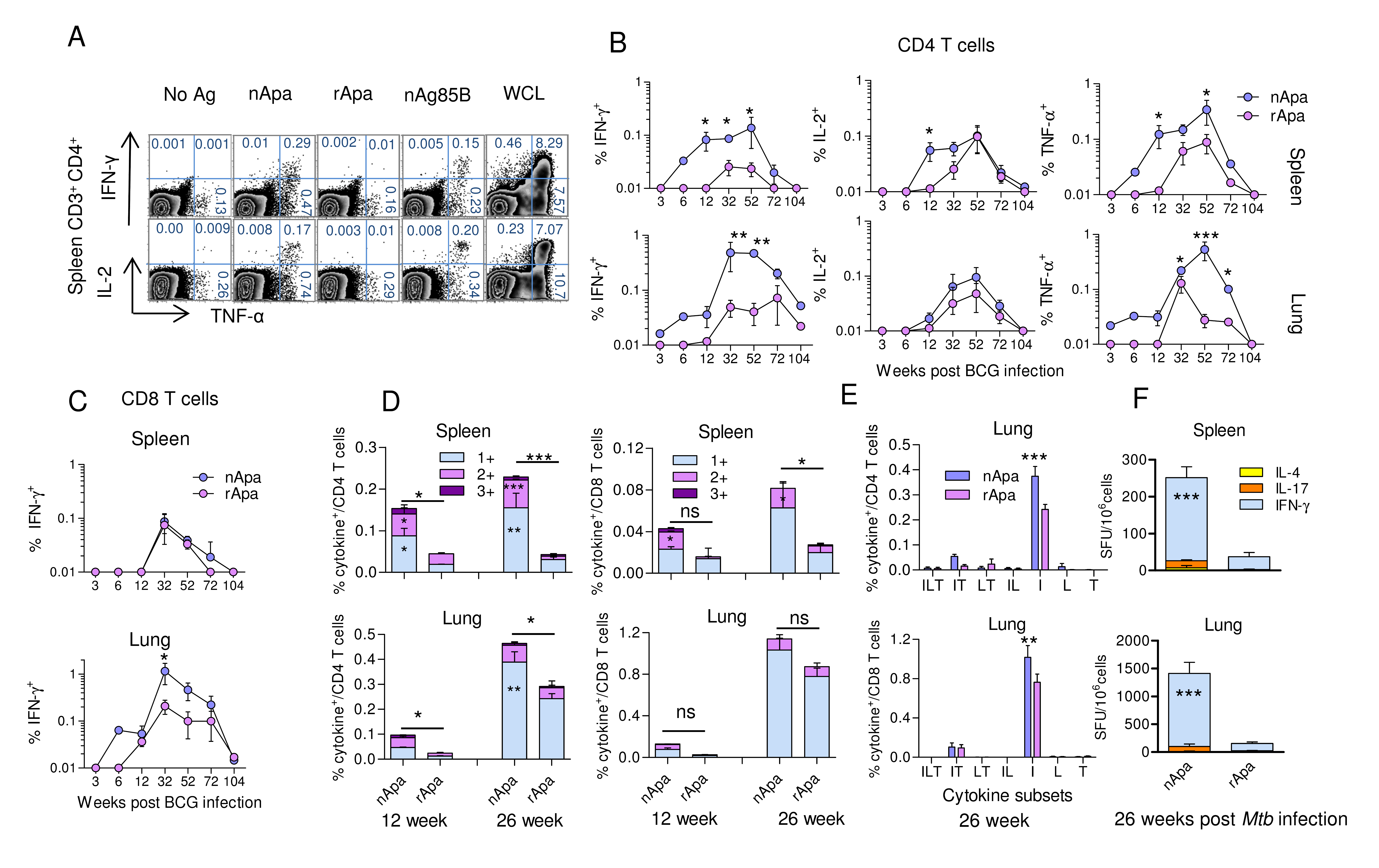

Supplement: Figure S2 — nApa is more antigenic than rApa in BCG and Mtb infected mice. (A–C) Mice were infected s.c. with 1×10^6^ CFU BCG, or (D–F) i.n. with 5×10^4^ CFU Mtb. At different time points after infection, as indicated, mice were euthanized and their spleen or lung cells were stimulated in vitro with no Ag, nApa, rApa, nAg85B or WCL. (A) The percentages (%) of TNF-α and IFN-γ (top) or IL-2 (bottom) producing cells among spleen CD4+ T cells from one representative experiment at the 52 wk time point are shown, and (B) the frequency (%) of nApa- or rApa-specific IFN-γ, IL-2 or TNF-α producing cells among CD4+ and (C) IFN-γ producing cells among CD8+ T cells from the spleen and lung at 7 different time points are plotted. Data at 12, 32, 52 and 72 wks (in B–C) are means ± s.e.m. of 3–4 independent mice experiments, while data (means) at 3, 6 and 104 wks are from one experiment using pooled cells (n = 4 mice) evaluated in duplicate. (D–F) T cell response in Mtb infected mice. (D) nApa- or rApa-specific IFN-γ, IL-2 and TNF-α cytokine co-expression profiles in the spleen and lung were determined at 12 and 26 wks after infection and the proportions of single (1+), double (2+), or triple (3+) cytokine producing T cell subsets constituting total cytokine positive (+) CD4+ or CD8+ T cells are plotted as % of CD4+ T cells (right) or CD8+ T cells (left), respectively. Total as well as individual subset responses are compared. (E) The percentages of 7 possible combinations of cytokine secreting CD4+ or CD8+ T cell subsets in the lung at 26 wks are also plotted. Data at 12 wks are means ± s.e.m. of pooled cell culture in triplicate while at 26 wks are of individual mice (n = 4). (F) nApa or rApa-specific IFN-γ, IL-17 or IL-4 SFU/106 spleen or lung cells at 26 wks. Data are means ± s.e.m. of triplicate or quadruplet cultures. * Significant using 1-way analysis of variance (ANOVA) followed by Bonferroni's multiple comparisons test (B–F). (TIF) [file ppat.1003705.s002.tif]

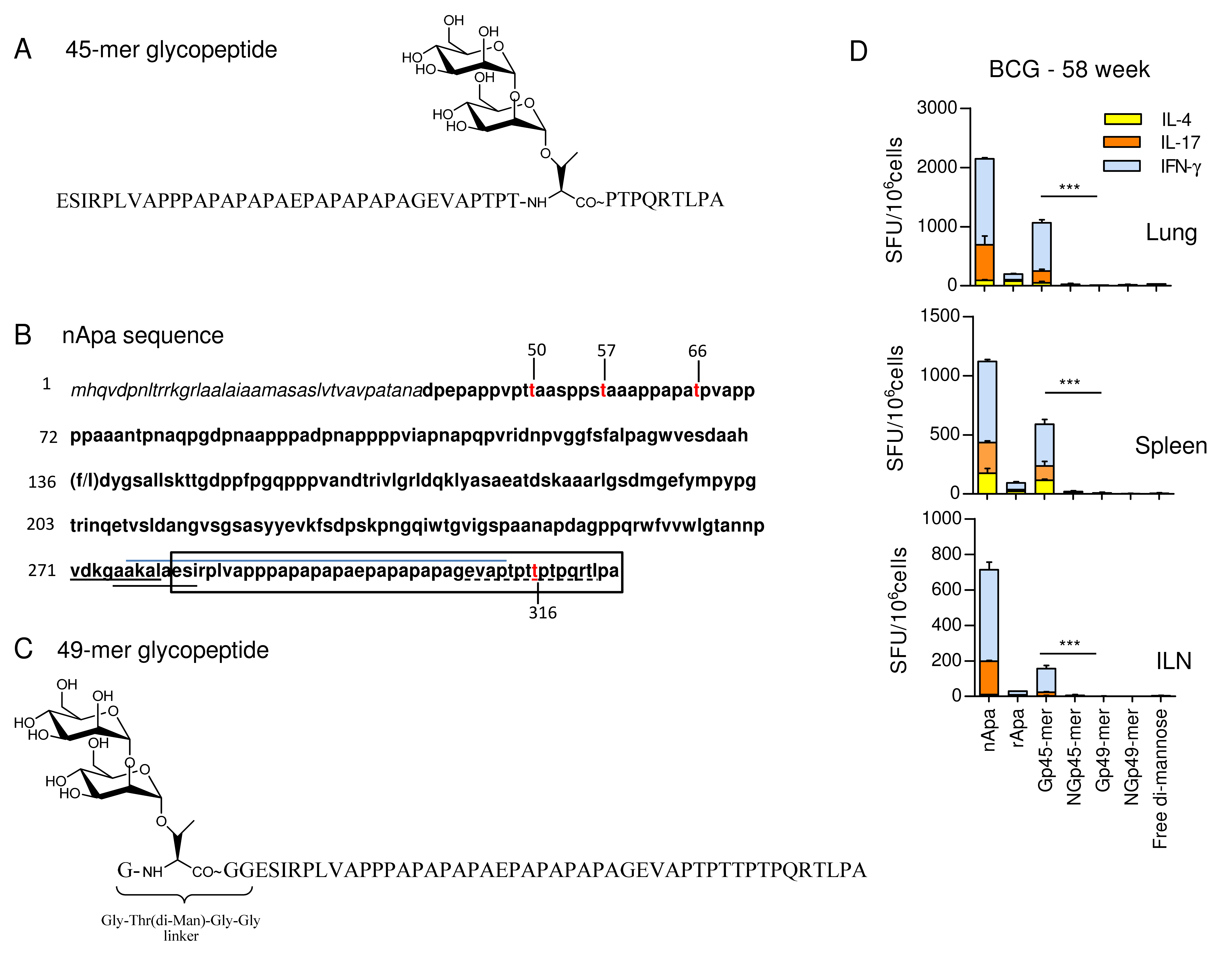

Supplement: Figure S3 — Amino acid sequence of Mtb Apa and synthetic C-terminal glycopeptides and antigenicity. (A) Structure of a synthetic 45-mer C-terminal Apa glycopeptide (residues 281–325). (B) Amino acid sequence of Apa. The signal peptide and the sequence of the protein are indicated in italic and bold-faced type, respectively. Parentheses indicate amino acid 137, phenylalanine for Mtb, and leucine for BCG. The sequence of synthetic 45-mer C-terminal glycopeptide and control non-glycopeptide (residues 281–325) evaluated in this study is indicated in the box. Threonine (Thr) residues at positions 50 and 57 are naturally glycosylated with mannobiose (α-D-Manp(1→2)α-D-Manp), the Thr residue at position 66 is modified with a single mannose (α-D-Manp), and Thr-316 is glycosylated with either a mannose, a mannobiose, or a mannotriose (α-D-Manp(1→2)α-D-Manp(1→2)α-D-Manp) (indicated in red color). The T cell epitope prediction analysis using IEDB program (http://www.immuneepitope.org/) indicated the probable binding of a 15-mer (residues 309–323) encompassing Thr316 to MHC class-II molecules encoded by H2I-Ad and H2E-Ad alleles, while peptide p271-288 is predicted to consist of 2 overlapping 9-mers with high affinity for MHC class-I molecules encoded by H2Kd allele (IEDB and http://www.syfpeithi.de/). The black solid line, dotted line and blue line indicates IEDB program predicted CD8+, CD4+ and B-cell epitopes (based on Kolaskar and Tongaonkar antigenicity) in Apa C-terminal domain (residues 271–325), respectively. (C) Structure of a synthetic 49-mer glycopeptide comprised of Apa peptide (residues 281–325, non-glycosylated at Thr-316) and N-terminal extension bearing dimannosyl-Thr residue linked to Apa 45-mer sequence via Gly-Gly dipeptide unit. (D) Synthetic 45-mer glycopeptide-, 49-mer glycopeptide- and free di-mannose-specific cytokine responses of BCG infected mice in ELISPOT assay. Stimulation with mature proteins (nApa or rApa) or non-glycopeptides (45-mer and 49-mer) was included [file ppat.1003705.s003.tif]

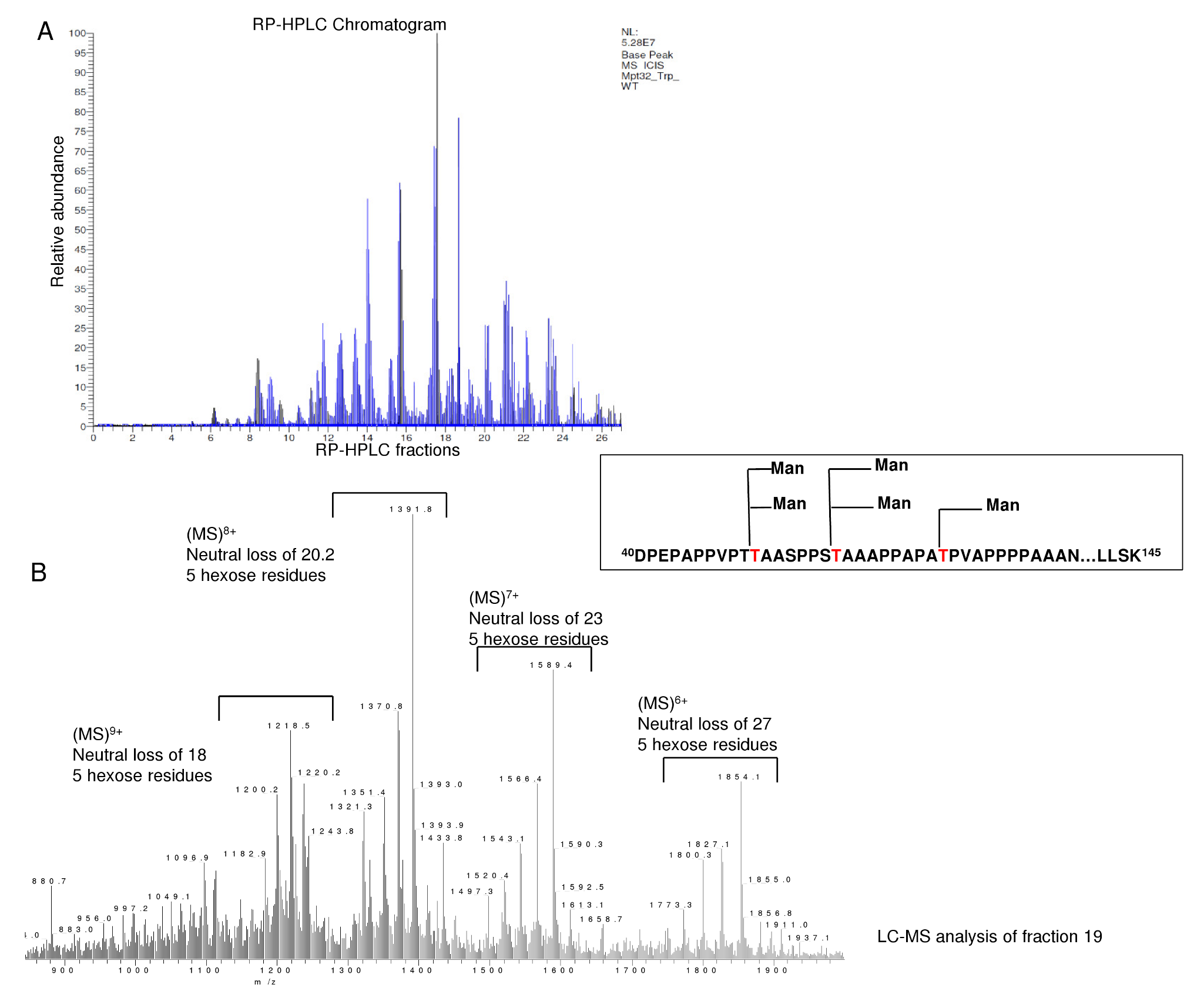

Supplement: Figure S5 — Fractionation of nApa trypsin-digest and LC-MS analysis of fractions. (A) The RP-HPLC fractionation of nApa trypsin-digest. A chromatogram with relative abundance of each fraction is shown. (B) LC-MS analysis of nApa trypsin-digest. A portion of each fraction was analyzed by LC-MS to identify the peptides represented by each fraction. Fraction 19 (shown), 21, and 22 (not shown) demonstrated the presence of the N-terminal glycopeptide for nApa, mannosylated with 5 (predominant) 4, or 3 residues. No other significant products were found in the most active fractions (19 and 22). The amino acid sequence of the N-terminal nApa glycopeptide is shown in the box. (TIF) [file ppat.1003705.s005.tif]

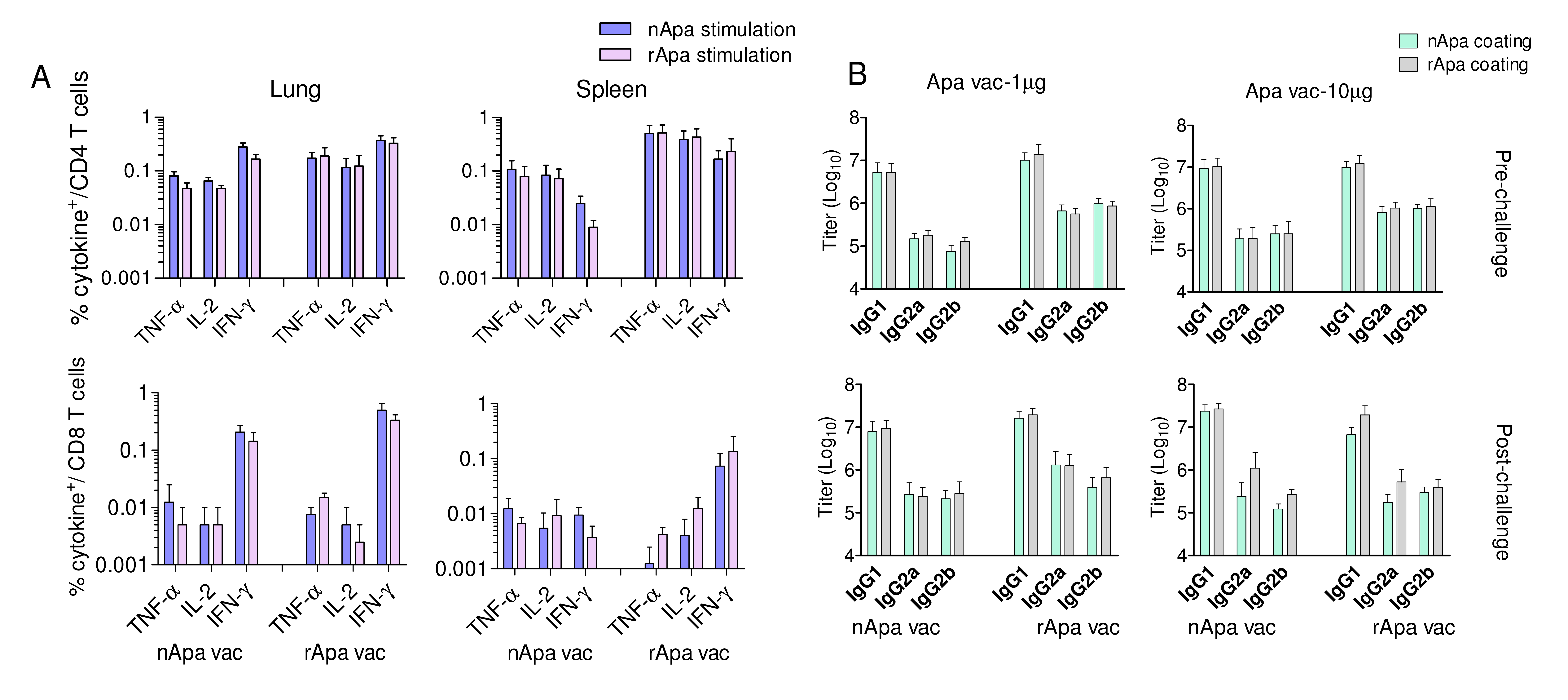

Supplement: Figure S6 — Both nApa and rApa are recognized equally well in Apa vaccinated and challenged mice. (A–B) Mice were vaccinated with nApa or rApa (either 1 or 10 µg/dose) in DDA-MPL adjuvant. Four wks after last vaccination dose, mice were challenged with Mtb as described in Figure 5. T and B cell responses to both nApa and rApa were investigated at the time of challenge and 6 wks post-challenge. (A) The frequency (%) of TNF-α, IL-2 or IFN-γ cytokine producing cells in the lung and spleen cells of Apa (1 µg/dose) vaccinated and challenged mice after in vitro stimulation with nApa or rApa are shown. Data are average response of 4 mice/group (± s.e.m.). (B) Anti-nApa and anti-rApa IgG1, IgG2a and IgG2b antibody titers in the sera of Apa vaccinated mice before and after challenge. Data are average response of 4 mice/group (± s.e.m.). (TIF) [file ppat.1003705.s006.tif]
