# Supplementary material for: O-mannosylation of the Mycobacterium tuberculosis Adhesin Apa Is Crucial for T Cell Antigenicity during Infection but Is Expendable for Protection
Source: PLoS Pathog. 2013 Oct 10;9(10):e1003705. doi: 10.1371/journal.ppat.1003705 (PMC3795050; doi:10.1371/journal.ppat.1003705)
Supplement: Text S1 — Supplementary information. Supplemental information includes details of peptide synthesis, ELISPOT assay, flow cytometry, trypsin proteolysis of Apa and RP-HPLC fractionation of digests, and Table S1. (DOCX) [file ppat.1003705.s007.docx]

**SUPPLEMENTAL INFORMATION**

***O*-mannosylation of the *Mycobacterium tuberculosis* Adhesin Apa is Crucial for T cell Antigenicity during Infection but is Expendable for Protection**

**Subhadra Nandakumar, Sunil Kannanganat, Karen M. Dobos, Megan Lucas, John S. Spencer, Sunan Fang, Melissa A. McDonald, Jan Pohl, Kristin Birkness, Venkateswarlu Chamcha, Melissa V. Ramirez, Bonnie B. Plikaytis, James E. Posey, Rama Rao Amara and Suraj B. Sable**

**Supplementary Materials and Methods**

**Peptide Synthesis**

The peptides were assembled using a combination of manual and automated (model Apex 396 synthesizer, AAPPTECH, Louisville, KY or model 433A synthesizer, Applied Biosystems, Foster City, CA) Fmoc solid phase synthesis (SPPS) [49]. The C-terminal 45-mer Apa (glyco) peptides (residues 281-325) and 49-mer (glyco) peptides were assembled starting with Fmoc-Ala-Wang-resin; the Fmoc-amino acids were incorporated using a double coupling protocol as their in-situ formed N-hydroxybenzotriazole esters in N-methylpyrrolidone (NMP). The Fmoc group was manually deprotected using three successive treatments with 20% (v/v) piperidine in NMP (5 min) prior to coupling of the next residue. In the Apa position 316 of the 45-mer glycopeptide and in position 2 of the 49-mer glycopeptide, Thr was manually incorporated as peracetylated Fmoc-Thr(α-Man-[1,2]-α-Man)OH (Sussex Research, Ottawa, Canada) followed by manual coupling of residues 315 through 312 (45-mer glycopeptide) or of the N-terminal Gly (49-mer glycopeptide). To confirm completeness of glycosylated Thr-316 incorporation, small portions of Fmoc-deprotected peptide [(312/315/316)-325]-resins were cleaved using TFA/phenol/thioanisole/ethanedithiol/water for 2 h at 25 ºC followed by precipitation with diethyl ether. The masses of the peracetylated intermediates were confirmed by MALDI-TOF MS (Table S1). The remaining Apa residues, 281-311, were incorporated by automated SPPS using a program that was optimized for the assembly of non-glycosylated 45-mer 281-325. The peracetylated glycopeptides were cleaved off/deprotected and precipitated as described above, and the O-acetyl groups were removed by treatment with hydrazine/methanol (1:4.5, v/v; 2.5 h at 25 ºC) [50] yielding a crude product. The peptides were purified by preparative RP-HPLC on a Zorbax SB-C18 column (1×25 cm, d_p_=5 um, 300Å pore size) eluted using a linear gradient of acetonitrile in 0.08% aqueous TFA. The masses of the final products were confirmed by MALDI-TOF MS (Table S1), their N-terminal sequence homogeneity was verified by automated Edman degradation, and the chromatographic purity (>95%) was confirmed by C18-RP-UHPLC. All peptides were obtained lyophilized in the form of their trifluoroacetate salts.

**ELISPOT Assay**

In brief, 96 well ELISPOT plates were coated with 100 µl of 5 µg/ ml respective capture Ab in PBS (pH 7.2) and incubated overnight at 4°C. Free binding sites were blocked with RPMI-1640 medium containing 10% fetal calf serum (FCS; Atlas Biologicals, Fort Collins, CO) for 2 h at room temperature. Human PBMCs or mouse lung, spleen and ILN cells isolated as described before [18,35,52] were suspended at 1 or 2 × 10^5^ cells per well in RPMI-1640 medium supplemented with 100 IU/ml penicillin, 50 µg/ml streptomycin, 1 mM L-glutamine, 25 mM HEPES, 1 mM sodium pyruvate, 50 µM β-mercaptoethanol, 1× vitamins and 1× nonessential amino acids (Gibco-Invitrogen, Grand Island, NY) and 10% heat-inactivated pooled healthy human AB^+^ serum for human PBMCs or FCS for mouse cells. Cells were stimulated with 10 µg/ml of nApa, rApa, WCL, STCF, ESAT-6, CFP-10 or synthetic peptides (unless mentioned otherwise) for 40 h. Stimulation with PHA or Con-A (1 µg/ml; Sigma-Aldrich, St. Louis, MO) was used as a positive control for cell viability and reactivity. After incubation at 37°C the wells were washed with PBS-Tween-20 and the site of cytokine secretion was detected with a biotin-labeled detection Ab and horseradish peroxidase-conjugated streptavidin. The enzyme reaction was developed using 3-amino-9-ethylcarbazole (AEC) substrate reagent set (BD-Biosciences, San Diego, CA). The number of spot forming units (SFU) per well were counted using an ELISPOT reader (Cellular Technology Limited, Cleveland, OH). The number of spots specific for each Ag preparation was calculated by subtracting the number of spots that formed in the absence of added Ag from the number that formed in its presence.

**Flow Cytometry**

Briefly, 2×10^6^ cells were dispensed in 96 well polypropylene plates in supplemented RPMI-1640 medium and stimulated with 10µg/ml of *Mtb* Ag in a total volume of 200 µl. Stimulation with PMA-ionomycin (1 µg/ml) was used as a positive control. Stimulations were conducted in the presence of anti-CD28 and anti-CD49d Abs (1 µg/ml; BD Pharmingen) and the cells were incubated at 37°C in the presence of 5% CO_2_ for 8 h. Brefeldin A (10 µg/ml) and monensin (3μM; BD pharmingen) was added and cells were cultured for additional 4 h. Cells were stained with surface and intracellular anti-human or anti-mouse Abs. Anti-mouse Abs (BD) for ICS were Pacific blue-anti-CD3(clone 500A2), Alexa Fluor 700-anti-CD4 (clone RM4-5), PerCP-anti-CD8 (clone53-6.7), APC-anti-IFN-γ (clone XMG1.2), PE-Cy7-anti-TNF-α (clone MP6-XT22) and PE-anti-IL-2 (clone JES6-5H4). Anti-human Abs (BD) for ICS were Pacific blue-anti-CD3 (SP34-2), PerCP-anti-CD4 (clone SK3), Amcyan-anti-CD8 (clone SK1), Alexa Fluor 700-anti-IFN-γ (clone B27), PE-Cy7-anti-TNF-a (clone MAb11) and APC-anti-IL-2 (MQ1-17H12). For DC activation, PBMCs were stimulated with 10µg/ml *Mtb* Ags for 12 h. Anti-human Abs used were Pacific blue-anti-CD3 (clone SP-34-2), Pacific blue-anti-CD14 (cloneM5E2), Alexa780-CD20 (clone 2H7), PerCP-anti-HLA-DR (clone G46-6), APC-anti-CD11c (clone S-HCL-3), PE-anti-CD123 (clone 7G3) and FITC-anti-CD80 (clone BB1). The stained cells were fixed with 1% paraformaldehyde and acquired on the LSRII system (BD Immunocytometry Systems) and analyzed using FlowJo software (Treestar, Inc., San Carlos, CA). Gating strategy as described previously [52] was used. Boolean combination gating for IFN-γ, IL-2 and TNF-α was performed to calculate the frequencies of expression profiles corresponding to the 7 different combinations of cytokines using FlowJo software. After subtracting the background values, frequencies of individual or total cytokine secreting CD4^+^ or CD8^+^ T cells were plotted for each Ag.

**Digestion of Apa with Trypsin and Separation of Digest Mixtures.**

Recombinant and native Apa were hydrated in digest buffer (0.5 M urea, 0.02 M methylamine, 0.05 M NaCl, and 0.001 M CaCl_2_ in 0.01 M Tris-HCl, pH 7.5) to 2.5 mg/ml. Trypsin (modified, sequencing grade, Roche Chemical Co. Indianapolis, IN) was added at an enzyme:substrate ratio of 1:70, and samples were incubated with gentle agitation at 37^0^C for 12 h. Additional trypsin was added at 12 h and samples incubated for an additional 6 h. The reaction was stopped by 3 freeze-thaw cycles and the digest mixtures were stored at -20^0^C until use.

Trypsin digested nApa or rApa was acidified by addition of 10% TFA to a final concentration of 0.5%. The digest was applied to a reversed phase C18 HPLC column (20 cm x 4.6 mm, Vydac Inc., Hyperia, CA) at 0.1 ml/min using an Alliance HPLC system (Waters, Inc., Bedford, MA). The column was washed with 5 column volumes of buffer A (0.1% TFA) at 0.5 ml/min and peptides eluted with a gradient of 100% buffer A to 100% buffer B (80% acetonitrile in buffer A) over 10 column volumes.

Whole digests of nApa, rApa and separated peptides were analyzed by liquid chromatography-mass spectrometry (LC-MS) using an LCQ Classic electro-spray ionization mass spectrometer (ES-MS, ThermoFinnigan, San Jose, CA) and a Bruker Daltonics Ultraflex matrix assisted laser desorption ionization time of flight mass spectrometer (MALDI-TOF-TOF, Bruker Daltonics Inc, Billerica, MA). For MALDI-TOF, 1 ml of sample was mixed with 1 ml of matrix (dihydroxybenzoic acid, 10 mg/ml in 50% acetonitrile, 0.1% trifluoroacetic acid) spotted on a MALDI target and allowed to air dry. The spectra were collected in positive ion reflector mode using a 25 kV accelerator voltage.

**Supplementary Table**

**Table S1: Masses of C-terminal 45-mer and 49-mer (Glyco) peptides and Intermediates**

| **Apa peptide (residues)** | **Mass**  **theoretical** | **Mass**  **found** |
| --- | --- | --- |
| Peracetylated T(di-Man)PTQRTLPA (316-325) | 1920.65 | 1920.85 |
| Peracetylated TT(di-Man)PTQRTLPA (315-325) | 2021.69 | 2021.91 |
| Peracetylated PTPTT(di-Man)PTQRTLPA (312-325) | 2318.18 | 2317.93 |
| Glycosylated 45-mer (281-325) | 4693.33 | 4693.34 |
| Non-glycosylated 45-mer (281-325) | 4366.33 | 4366.51 |
| Glycosylated 49-mer GT(di-Man)GG(281-325) | 4962.55 | 4962.56 |
| Non-glycosylated 49-mer GTGG(281-325) | 4638.44 | 4638.58 |
